# Bridging knowledges through fire: a systematic review of indigenous fire management practices in Brazil

**DOI:** 10.1186/s13002-026-00884-7

**Published:** 2026-04-14

**Authors:** Caique Vasconcelos Dantas, Eraldo Medeiros Costa Neto, Elmo Borges de Azevedo Koch, Fabio de Oliveira Roque

**Affiliations:** 1 Programa de Pós-Graduação em Ecologia e Evolução, Universidade Estadual de Feira de Santana, Feira de Santana, BA Brazil; 2 Universidade Federal de Mato Grosso do Sul, Campo Grande, MS Brazil

**Keywords:** Biocultural resilience, Traditional Ecological Knowledge (TEK), Landscape restoration, Adaptive governance, fire-dependent landscapes

## Abstract

Despite increasing attention following recent catastrophic wildfires in Brazil, research on Indigenous Fire Management remains fragmented across regions and disciplines. This study performed a systematic review, following PRISMA 2020 guidelines, to integrate evidence on IFM across Brazilian biomes, practices, and governance contexts. Searches in major databases (1970–2025) yielded 605 records, of which 20 met eligibility criteria. The studies document 95 Indigenous Territories with systematic fire use, mainly in the Cerrado (52%) and Amazon (36%), followed by the Pantanal (7%) and Atlantic Forest (5%). Fire practices were categorized into five functional axes: agricultural (swidden and fallow enrichment), hunting and fruiting (patch-mosaic burning), cultural and ritual (localized low-intensity burns), prevention and territorial defense (prescribed burns, firebreaks, brigades), and ecological/restorative (control of invasive grasses and maintenance of clearings). Evidence indicates that Indigenous-managed fires enhance landscape heterogeneity, reduce fuel continuity, and significantly lower the extent and severity of uncontrolled wildfires, while strengthening food security and territorial governance. Policy analyses reveal a gradual transition from restrictive “zero-fire” policies toward adaptive co-management models led by the Prevfogo/Ibama program and recognition of Indigenous Fire Stewardship. Overall, IFM emerges as a biocultural conservation strategy that aligns ecological dynamics with traditional knowledge and governance systems, offering promising pathways for climate adaptation and ecosystem restoration. Yet, challenges persist, including unstable funding and limited operational support for Indigenous brigades outside the dry season.

## Introduction

Wildfire is a central element in the ecological and cultural history of tropical ecosystems, playing an ambivalent role as both a disturbance agent and a driver of renewal [59]. In savanna environments such as the Cerrado, fire constitutes a key ecological process structuring landscapes, vegetation dynamics, and nutrient cycles [9, 57]. In contrast, in humid forests like the Amazon, uncontrolled or intensified burning poses a severe threat to biodiversity and ecosystem resilience. Within this context, traditional fire management practiced by Indigenous peoples and local communities emerges as an ancient form of ecological engineering [4], capable of shaping habitat mosaics, promoting biological diversity, and ensuring food and territorial security [39, 47, 49, 74].

Recent studies have demonstrated that Indigenous Lands (ILs) in Brazil play a strategic role in socioecological conservation, not only for protecting approximately 13% of the national territory [28], but also for acting as effective buffers against deforestation and large-scale wildfires. Throughout this manuscript, we use the term Indigenous Lands (ILs) to refer to legally recognized areas traditionally occupied by Indigenous peoples in Brazil, as defined by the 1988 Federal Constitution. Although the concept of “territory” may encompass broader sociocultural and cosmological dimensions, we adopt the legal terminology to ensure consistency and clarity. These territories function as effective ecological barriers to the expansion of agricultural frontiers [23, 51, 53, 60, 68, 72]. In agribusiness frontiers such as Mato Grosso and the Xingu region, Indigenous territories often serve as *fire refuges*, where traditional and managed burning contrasts with uncontrolled fires, resulting in lower rates of deforestation and biomass loss [60, 68, 72]. Far from being random, Indigenous fire management follows ecological and social calendars based on astronomical, climatic, and biological indicators, including the onset of rainfall, the flowering of indicator species, and wind direction [23, 26, 47]. The conceptual distinction between *“good fire”* (controlled and beneficial) and *“bad fire”* (uncontrolled and destructive), present among groups such as the Xerente and Krahô, exemplifies the sophistication of these regimes, in which timing, intensity, and spatial extent determine ecological and cultural outcomes [47, 76].

Fire management practices in Brazil exhibit substantial variation according to biome, livelihood strategies, and cosmological understanding ([47]; [23, 26]). In the Amazon, fire use tends to be limited, primarily applied for opening shifting cultivation fields, enriching fallows, and protecting villages perimeters [37, 51, 55]. In contrast, in the Cerrado and ecotonal regions, fire is used more frequently and systematically, associated with hunting, fruiting cycles, and the control of dry biomass accumulation [47, 53]. Notable examples include the Krahô of Tocantins, who maintain a mosaic burning regime for hunting and fruit management [47]; the Xavante and Paresi of Mato Grosso, who have reintroduced traditional techniques through partnerships with Indigenous fire brigades coordinated by Prevfogo/Ibama [28]; and the Pemon of the Gran Sabana in Venezuela, whose ritual and ecological use of fire offers a regional parallel to Brazil’s recent intercultural initiatives in fire governance [49].

Despite its ecological and cultural relevance, Indigenous fire knowledge has historically been marginalized by “zero-fire” conservation policies rooted in Eurocentric and colonial ecological paradigms that overlooked the regenerative role of cultural burning [23, 31]. This exclusionary paradigm began to shift in the 2010s with the emergence of Integrated Fire Management (IFM), which integrates ecological science, adaptive management, and local knowledge, recognizing fire as a legitimate ecological and cultural process [8, 22, 28, 67]. In Brazil, IFM programs have reduced fire incidence by more than 40% in Indigenous Territories, while revitalizing traditional ecological calendars and generating local employment opportunities [27]. The Indigenous Federal Fire Brigades coordinated by *Prevfogo/Ibama* exemplify this paradigm shift, demonstrating that integrating traditional knowledge with technical management can reduce the frequency of severe fires and strengthen territorial autonomy [23, 53].

In the Brazilian Cerrado, Pivello [56] demonstrated that early dry-season, low-intensity burns are ecologically compatible with fire-adapted vegetation, preventing megafires and sustaining biodiversity. The Brazilian case is particularly relevant because it integrates one of the world’s greatest sociocultural and ecological diversities, offering a living laboratory for understanding the coevolution of fire, biodiversity, and culture [4, 74]. Understanding and systematizing Indigenous fire management practices in Brazil, considering their regional variation, cosmological foundations, and contemporary challenges, is therefore crucial for developing inclusive, intercultural, and effective environmental policies [23, 28, 49]. Intercultural and participatory fire governance remains essential yet insufficiently implemented across South American contexts such as Brazil, Guyana, and Venezuela [8, 48].

Despite the growing scholarly attention catalyzed by recent catastrophic wildfire events in Brazil, research on fire ecology and Indigenous fire management remains fragmented and dispersed across disciplines, regions, and methodological approaches. Studies often differ in scale, terminology, and focus—ranging from ecological assessments of fire regimes to ethnographic analyses of cultural burning—making it difficult to synthesize knowledge or inform coherent policy frameworks. Moreover, much of the literature is scattered across gray reports, governmental documents, and locally published works that are rarely integrated into global fire governance debates. A bibliometric review is therefore necessary to systematically map this expanding body of knowledge, identify conceptual gaps and research trends, and clarify how scientific and Indigenous perspectives have converged or diverged over time. Such an analysis can reveal the evolution of fire-related research in Brazil, highlight emerging interdisciplinary collaborations, and support the development of a more integrated and inclusive agenda for fire governance and biocultural conservation. It is important to note that we acknowledge that our perspectives are shaped by academic traditions with predominantly Eurocentric historical roots, even though we were raised and trained in diverse regions of Brazil. While many of us have engaged in collaborative initiatives with traditional and Indigenous communities, we recognize that the views reported here do not capture the full spectrum of lived experiences, worldviews, and cosmologies that are associated with the use of fire by their communities.

Accordingly, this study aims to identify the Indigenous communities in Brazil that actively employ fire as part of their territorial management practices, specifying the states and biomes in which they are located and the particular techniques they use. Furthermore, it examines the ecological, cultural, and political implications of these practices through an integrated biocultural sustainability framework, highlighting how traditional fire management contributes to biodiversity conservation, climate resilience, and the reinforcement of Indigenous territorial governance.

In this manuscript, we distinguish between Indigenous fire management and Integrated Fire Management (IFM). Indigenous fire management refers to traditional, culturally embedded fire practices developed and maintained by Indigenous peoples, grounded in cosmological, ecological, and territorial knowledge systems [7, 24, 49]. These practices are often described as cultural burning or Indigenous fire stewardship and are embedded in long-term socioecological relationships with the land [41, 49]. In contrast, Integrated Fire Management (IFM) refers to a policy-oriented and institutional framework that combines fire prevention, prescribed burning, suppression, and monitoring strategies within coordinated governance systems [50]. While these approaches may intersect in practice, particularly where Indigenous knowledge is incorporated into state fire policies, they emerge from distinct epistemological and governance frameworks [24, 50].

## Materials and methods

This systematic literature review was conducted in accordance with the PRISMA 2020 (Preferred Reporting Items for Systematic Reviews and Meta-Analyses) guidelines [54]. The protocol was not previously registered, as the study does not involve clinical or health-related data.

1. **Eligibility criteria**

This review intentionally restricted inclusion to peer-reviewed empirical publications in order to ensure methodological consistency, analytical comparability, and replicability of the evidence base.

We considered peer-reviewed scientific publications, including journal articles and academic book chapters, published between 1970 and 2025, that directly addressed the use, management, governance, or ecological implications of fire by Indigenous peoples in Brazil.

The inclusion criteria were as follows: (i) empirical studies describing traditional, cultural, ecological, or governance-related fire-use practices; (ii) publications written in Portuguese or English; (iii) explicit identification of the Indigenous community or ethnic group studied; (iv) clear indication of the territorial context (state and biome within Brazil). We excluded: (i) technical reports and institutional documents prepared by governmental or non-governmental organizations; (ii) theses, dissertations, and other non-peer-reviewed works; (iii) purely conceptual or theoretical studies without empirical data; (iv) studies addressing fire use in non-Indigenous contexts; (v) studies conducted outside Brazilian territory.

Although grey literature (e.g., theses, institutional reports) may contain relevant information, it was excluded to preserve a transparent, standardized, and reproducible corpus of indexed peer-reviewed evidence. Consequently, this review synthesizes documented patterns within the retrievable academic literature rather than attempting to exhaustively represent all Indigenous fire practices in Brazil.

(2) **Information sources**

The bibliographic search was conducted in both national and international databases, including Web of Science, Scopus, SciELO, and Google Scholar. These platforms collectively provide extensive coverage of environmental sciences, ethnobiology, anthropology, and socioecological research, and are widely used in systematic reviews [12]. Database searches were conducted between January and March 2025.

(3) **Search strategy**

Search descriptors were developed in both Portuguese and English and combined using Boolean operators (AND, OR), following recommended procedures for systematic reviews [14].

The use of bilingual descriptors reflects the linguistic structure of academic production on Indigenous fire management in Brazil. A substantial portion of empirical research conducted in Brazilian Indigenous territories is published in Portuguese, particularly in national journals and regional outlets indexed in SciELO and Google Scholar. Conversely, studies published in international journals are typically indexed and retrieved through English-language descriptors in databases such as Web of Science and Scopus. Therefore, employing both Portuguese and English search terms was necessary to maximize retrieval sensitivity and reduce language-based selection bias.

The primary search combinations were: (i) (“manejo do fogo” OR “queima controlada” OR “fogo indígena”) AND (“povos indígenas” OR “comunidades tradicionais”); (ii) (“indigenous fire management” OR “traditional fire practices” OR “indigenous fire stewardship”) AND (“Brazil”).

Although the descriptors were not literal translations of each other, they were designed to capture semantically equivalent concepts within distinct indexing and publication contexts. Portuguese terms tend to reflect locally used expressions in ethnographic and policy-oriented literature, whereas English descriptors are more common in international fire ecology and governance studies.

To assess potential implications of using different descriptors, search results were cross-checked across databases to identify overlapping records and ensure consistency in retrieved themes. Duplicate records were removed during the screening process. The use of bilingual descriptors increased initial sensitivity of the search while the subsequent eligibility screening ensured thematic specificity and methodological coherence.

Accordingly, the combined strategy aimed to balance comprehensiveness and reproducibility while minimizing linguistic bias in the identification of relevant peer-reviewed studies.

Search strings were adapted to the indexing syntax and search interfaces of each database. Filters were applied to restrict results to the defined publication period and language criteria.

(4) **Selection process**

**Fig. 1:**
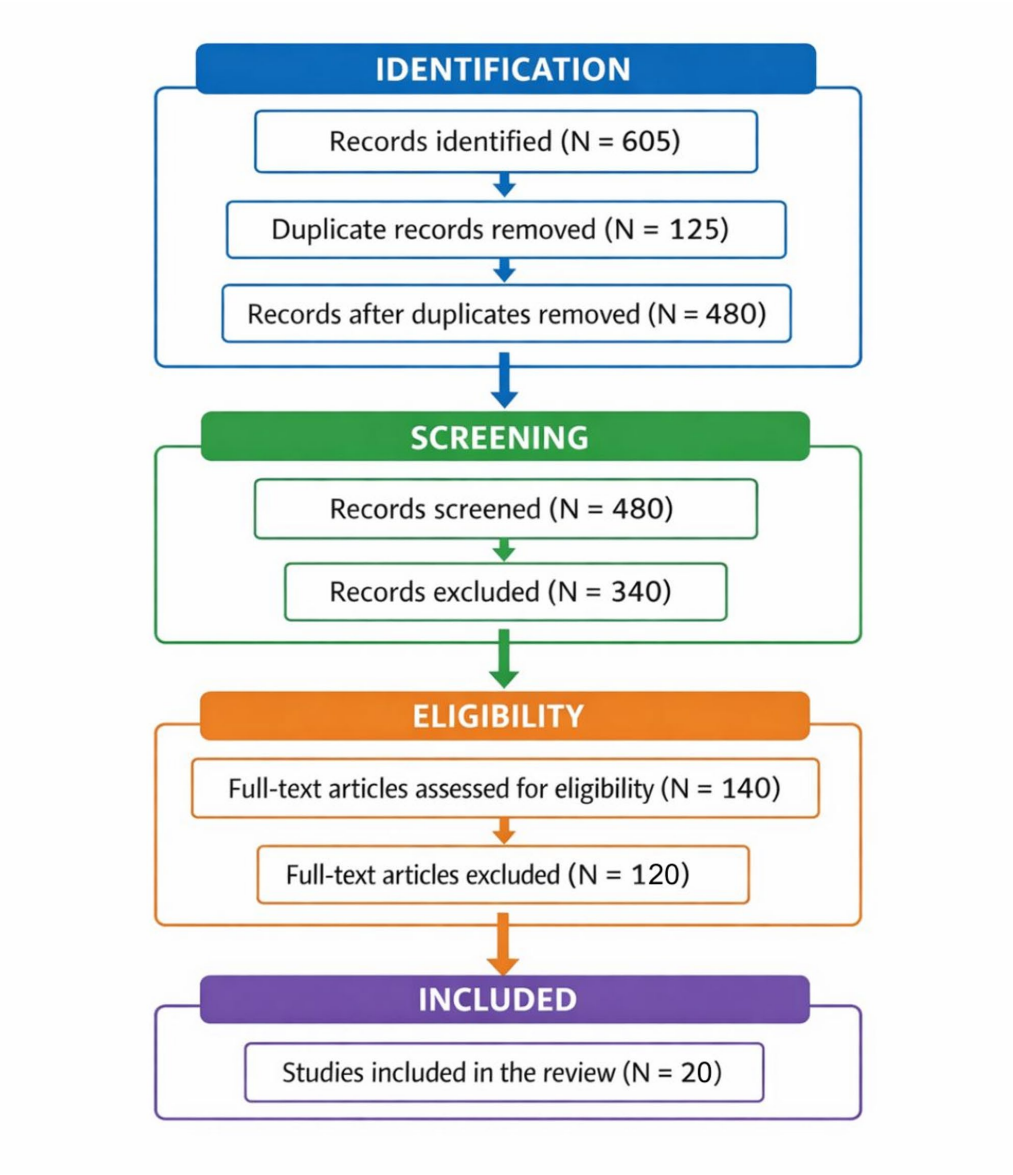
Flowchart depicting the study selection process

The screening process followed PRISMA 2020 guidelines and consisted of three stages: Identification; Title and abstract screening; Full-text eligibility assessment. During the identification phase, a total of 605 records were retrieved: 180 from Web of Science, 210 from Scopus, 120 from SciELO, and 95 from Google Scholar. After removing 125 duplicates, 480 unique records remained. During the title and abstract screening stage, 340 records were excluded for not meeting the eligibility criteria (e.g., non-Indigenous contexts, studies conducted outside Brazil, lack of empirical data). A total of 140 studies were retained for full-text review. During the eligibility phase, 119 studies were excluded for being exclusively theoretical, lacking clear territorial identification, or addressing fire management in comparative international contexts without direct empirical focus on Brazil. In total, 20 studies met all inclusion criteria and were retained for qualitative synthesis. The complete selection process is illustrated in a PRISMA 2020 flow diagram (Fig. 1).

(5) **Data collection**

From each included study, we extracted the following information: (i) Indigenous community or ethnic group studied; (ii) state and biome of occurrence; (iii) Indigenous Land or territory when specified; (iv) descriptions of fire management practices and the contexts in which fire was used; (v) functional purposes associated with these practices as described in the studies (e.g., agriculture, hunting/fruiting, ritual, prevention/defense, restoration); (vi) ecological and/or social outcomes reported; and (vii) year and type of publication.

All extracted information was compiled into a structured comparative spreadsheet, which served as the empirical basis for subsequent spatial, temporal, and thematic analyses of Indigenous fire management practices documented in Brazil.

(6) **Data analysis**

The compiled dataset was analyzed through three complementary analytical approaches.

First, a spatial analysis was conducted to examine the geographical distribution of studies across Brazilian states, biomes, Indigenous Lands, and ethnic groups. This analysis allowed us to identify patterns in the regional concentration of research and the territories where Indigenous fire practices have been documented.

Second, a temporal analysis was performed to evaluate the evolution of scientific publications over time, identifying periods of increased research attention and the overall trajectory of academic interest in Indigenous fire management in Brazil.

Third, we conducted a qualitative thematic analysis of the cultural, ecological, and social contexts associated with fire use described in the selected studies. Through an inductive coding process based on the explicit purposes of fire reported in each study, recurrent functional purposes of fire use were identified.

From this analytical process, five functional axes emerged: (1) agricultural; (2) hunting and fruiting; (3) cultural/ritual; (4) prevention and territorial defense; and (5) ecological/restorative.

These axes should not be interpreted as mutually exclusive categories. A single Indigenous Land may simultaneously exhibit multiple uses of fire depending on seasonal, ecological, and social contexts. During data coding, each documented practice was classified according to its stated function. When multiple purposes were described for the same territory, all relevant axes were assigned.

Therefore, the resulting figures represent the frequency of occurrence of functional purposes across territories, rather than exclusive or definitive typologies of Indigenous fire management. The operational definitions and scope of each functional axis are summarized in Table 1.

**Table 1:** Definition of the five functional axes of Indigenous fire use identified in the systematic review

Functional Axis	Operational Definition	Examples of Practices Documented
Agricultural	Fire used for cultivation cycles, soil fertilization, vegetation clearing, agroforestry management	Swidden (coivara), field preparation, ash enrichment
Hunting and Fruiting	Fire used to attract fauna, stimulate fruiting, manage habitat mosaics	Early dry-season mosaic burns, hunting expeditions
Cultural/Ritual	Fire embedded in cosmological, ceremonial, identity-based practices	Initiation rituals, symbolic burns
Prevention and Territorial Defense	Fire used to reduce fuel load, create firebreaks, protect villages	Prescribed burns, Indigenous brigades
Ecological/Restorative	Fire applied to promote regeneration, control invasives, maintain ecosystem structure	Grassland renewal, invasive species control

These categories were defined through an inductive analytical process based on recurring patterns identified during data extraction from the included studies. Rather than being pre-established a priori, the axes emerged from thematic convergence across the reviewed corpus. Each axis corresponds to a distinct functional purpose attributed to fire within Indigenous territories as documented in the empirical literature.

When a study reported multiple purposes for fire use within the same Indigenous Lands, multiple functional classifications were assigned, reflecting the overlapping and multifunctional nature of documented practices. The categorization was designed as an analytical synthesis tool to enable comparative assessment across territories and biomes, rather than as a rigid typology.

## Results and discussion

The empirical foundation of this section is restricted to the 20 studies that met the eligibility criteria established in the systematic review. Throughout the Results and Discussion, we explicitly distinguish evidence derived from the included corpus from contextual literature used for theoretical and ecological framing. This distinction ensures that the patterns, functional classifications, and governance interpretations presented below are directly supported by the systematically reviewed evidence base.

The 20 included studies span the period from 1978 to 2025 and represent distinct analytical phases in the documentation of Indigenous fire management in Brazil. Foundational ethnographic and historical accounts [13, 20, 33, 35, 43, 52, 58, 64–66] primarily describe agroecological and cosmological dimensions of fire use. A second group of studies [26, 47, 73] emphasizes mosaic burning and moral distinctions between controlled and uncontrolled fires in Cerrado landscapes. More recent publications [10, 18, 25, 28, 32, 53] focus on Indigenous Fire Brigades, Integrated Fire Management (IFM), and hybrid governance arrangements combining local ecological knowledge and institutional frameworks.

### Temporal trends in scientific production on indigenous fire management (1970–2025)

**Fig. 2:**
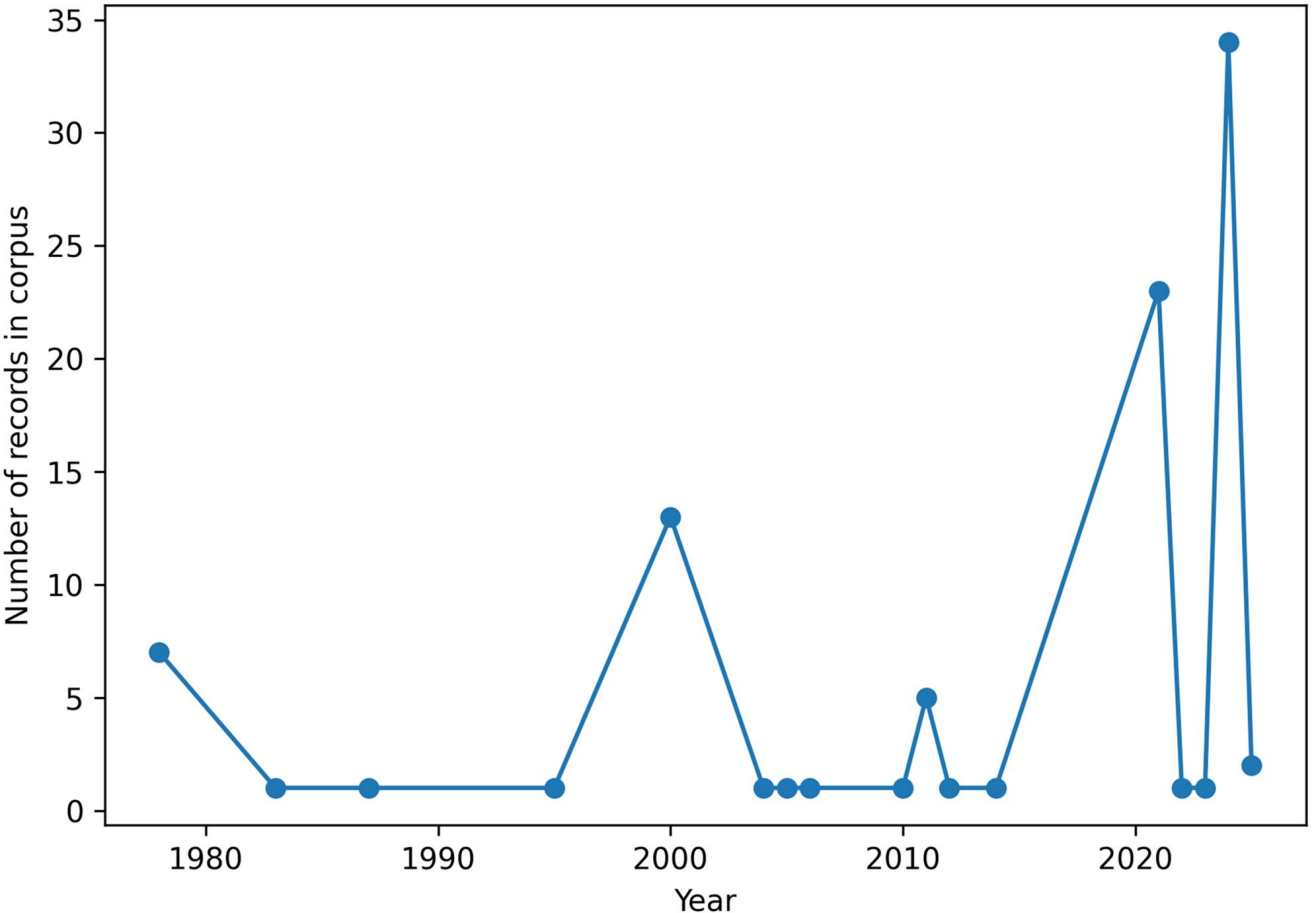
Temporal distribution of scientific publications addressing Indigenous fire management in Brazil (1978–2025)

The temporal distribution of the 20 included studies reveals a progressive increase in scientific production on Indigenous fire management in Brazil over the past five decades. Between 1970 and 1990, publications were sparse and primarily ethnographic in orientation, focusing on agroecological and cosmological dimensions of fire use (e.g. [13, 33, 58]), (Fig. 2).

From 1990 to 2010, the number of studies gradually expanded, incorporating ecological and landscape perspectives and engaging more directly with fire ecology debates (e.g. [47, 64, 66]), . This period coincides with broader discussions surrounding fire suppression policies and savanna ecology in Brazil.

A marked increase in publications occurs after 2010, particularly between 2015 and 2025, when governance-oriented and Integrated Fire Management (IFM) studies become more prominent (e.g. [10, 18, 25, 28, 32, 53]), . This temporal expansion aligns with the institutionalization of Indigenous Fire Brigades under the Prevfogo/Ibama program (established in 2013), intensified climate debates, and heightened public and political attention to extreme wildfire events in the Amazon and Cerrado.

Rather than indicating the recent emergence of Indigenous fire knowledge, the observed increase reflects a growing academic and institutional recognition of longstanding practices, now reframed within biodiversity conservation and climate adaptation agendas.

### General overview

**Fig. 3:**
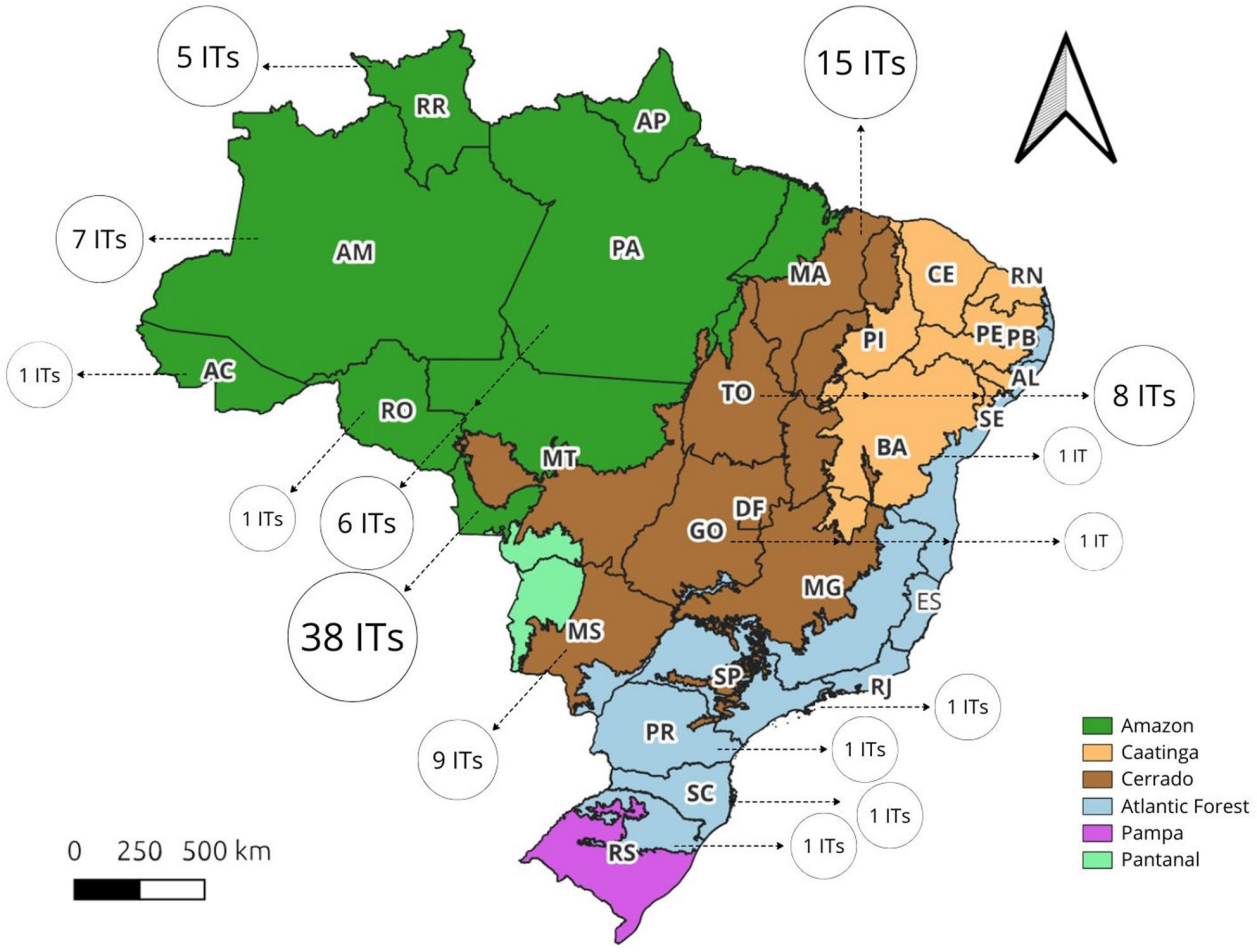
Map representing the distribution of Indigenous Lands (ILs) in Brazil where traditional fire management practices are documented, organized by state and biome. Circle size denotes the number of Indigenous territories per state. Map adapted from Dantas et al., [17]. State abbreviations are as follows: AC – Acre, AM – Amazonas, AP – Amapá, BA – Bahia, CE – Ceará, DF – Federal District, GO – Goiás, MA – Maranhão, MG – Minas Gerais, MS – Mato Grosso do Sul, MT – Mato Grosso, PA – Pará, PB – Paraíba, PE – Pernambuco, PI – Piauí, PR – Paraná, RJ – Rio de Janeiro, RN – Rio Grande do Norte, RO – Rondônia, RR – Roraima, RS – Rio Grande do Sul, SC – Santa Catarina, SE – Sergipe, SP – São Paulo, and TO – Tocantins

Based strictly on the 20 studies included in this systematic review, we identified 95 Indigenous Territories where fire management is empirically documented. Our analysis shows a predominant focus on the Cerrado (52%) and Amazon (36%) biomes (Fig. 3).

The predominance of Cerrado territories in the reviewed corpus is largely associated with studies focusing on Xavante, Krahô, Xerente, Paresi, and Kadiwéu territories [18, 28, 47, 53, 73], while Amazonian documentation is primarily derived from Kayapó, Munduruku, Yanomami, and Desana territories [10, 33, 43, 58, 65]. Pantanal cases are predominantly documented in studies addressing Kadiwéu, Guató, and Terena territories [10, 53], whereas Atlantic Forest cases are represented in research on Pataxó and Avá-Guarani territories [28, 43].

Within the reviewed corpus, fire practices were most frequently associated with agricultural use (15 studies: [10, 13, 20, 25, 26, 28, 32, 33, 35, 43, 52, 58, 64–66]), followed by hunting/fruiting (12 studies), prevention/territorial defense (10 studies), ecological/restorative functions (10 studies), and cultural/ritual uses (9 studies).

**Fig. 4:**
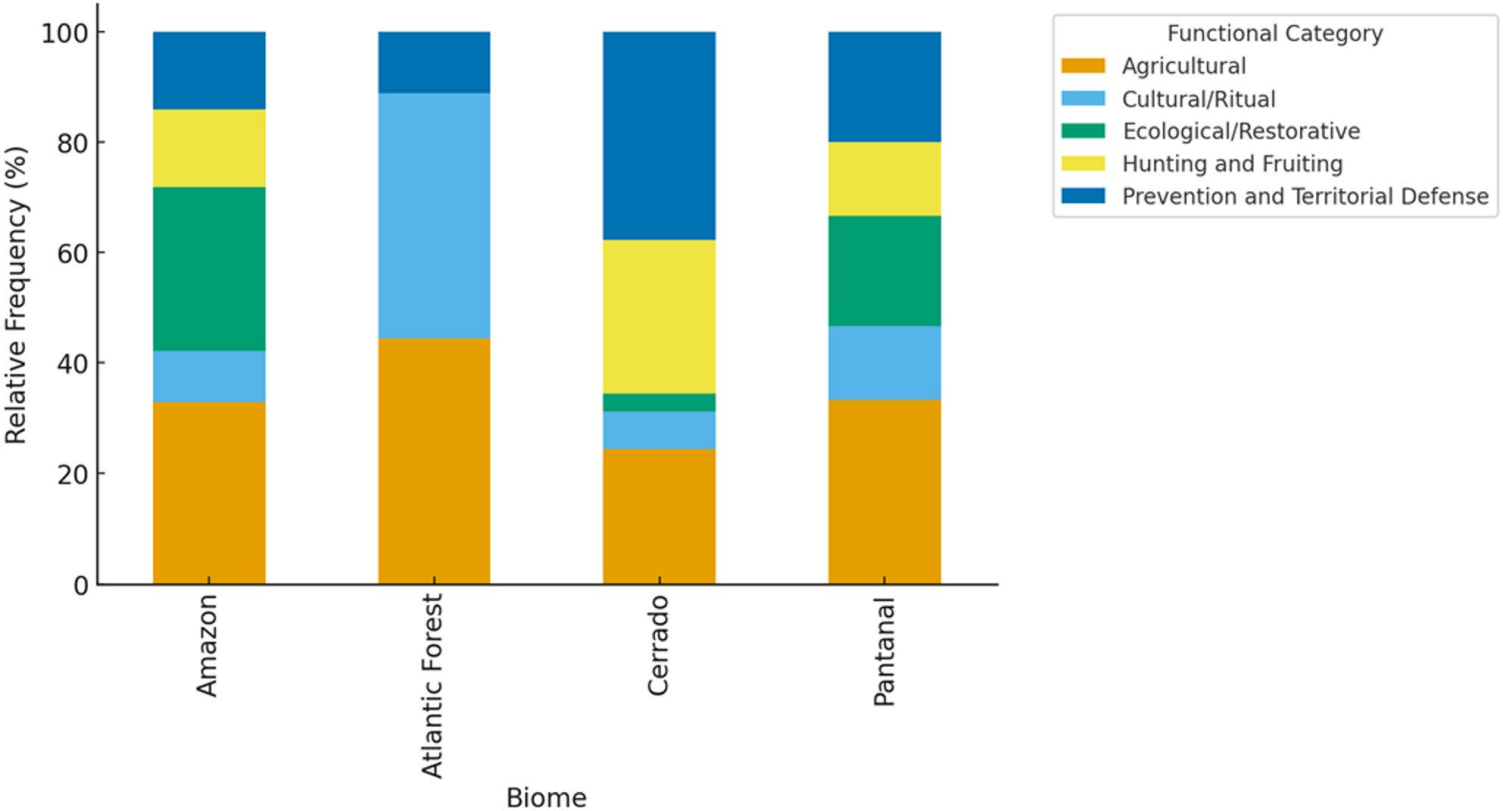
Functional axes represent non-exclusive categories; multiple axes may co-occur within the same Indigenous Lands

The distribution of these functional axes across biomes is detailed in Fig. 4; Table 1, which constitute the empirical basis of the interpretations presented below.

### Distribution of research across indigenous territories

Our systematic analysis of the 20 included studies reveals that they collectively report 95 Indigenous Lands (ILs). However, research attention is not evenly distributed across them. A substantial proportion of territories appear in only one publication, whereas a smaller subset is repeatedly documented across multiple studies.

Territories such as Xavante, Kayapó, Kadiwéu, Paresi, Krahô, and Guajajara appear in three or more publications, indicating sustained academic engagement and long-term research continuity. In contrast, the majority of Indigenous Territories are represented in a single study within the reviewed corpus.

This pattern reveals a marked concentration of research in a limited number of territories and ethnic groups, particularly those located in the Cerrado and Pantanal regions and those involved in Integrated Fire Management (IFM) programs. Five recent multi-territory studies account for a considerable proportion of the documented territories, reinforcing the presence of structural asymmetries in scientific production.

Importantly, this uneven distribution reflects patterns of research infrastructure, funding continuity, and institutional partnerships rather than the geographic distribution of Indigenous fire knowledge itself. The concentration of publications in certain territories should therefore not be interpreted as indicating greater prevalence of fire management practices in those regions, but rather greater academic visibility.

All territorial distribution analyses presented here derive exclusively from the 20 studies included in the systematic review (Table 2).

### Functional axes of fire use

All extracted information was organized into a structured comparative spreadsheet, which served as the basis for descriptive and comparative analyses of Indigenous fire practices documented in the reviewed literature. Through this analytical process, documented practices were classified into five functional axes: (1) agricultural; (2) hunting and fruiting; (3) cultural/ritual; (4) prevention and territorial defense; and (5) ecological/restorative.

These categories were defined through an inductive analytical process based on recurring patterns identified during data extraction from the included studies. Rather than being established a priori, the axes emerged from thematic convergence across the reviewed corpus, reflecting the diversity of cultural, ecological, and social purposes attributed to fire within Indigenous territories as documented in the empirical literature.

Importantly, these axes are analytical categories rather than mutually exclusive classifications. A single Indigenous Land may simultaneously exhibit agricultural, hunting, restorative, ritual, and preventive uses of fire depending on seasonal, ecological, and social contexts. During data extraction, each documented fire practice was coded independently according to its explicitly described function in the source study. When a study reported multiple purposes for fire within the same territory, all relevant axes were assigned.

Therefore, the resulting classification reflects the diversity of documented functions rather than exclusive typologies. The figures derived from this categorization represent the frequency of occurrence of functional purposes across territories, rather than isolated or definitive classifications of Indigenous fire regimes. The operational definitions and scope of each functional axis are summarized in Table 1.

The five functional axes identified in this review emerged from recurring patterns in the extracted data (Table 1). Several Indigenous Lands exhibit more than one functional category, indicating overlapping ecological and sociocultural uses of fire. Approximately 60% of the documented Indigenous Lands are associated with two or more functional axes, reinforcing the multifunctional character observed in the corpus.

Agricultural fire use dominates all regions, particularly in the Cerrado and Pantanal, reflecting the integration of burning with shifting cultivation, native pasture management, and biomass control. In contrast, cultural and ritual fires are more frequent in the Atlantic Forest, while ecological and restorative uses are relatively prominent in the Amazon and Pantanal, where they contribute to secondary forest regeneration and wetland maintenance. Preventive and territorial defense fires are most significant in the Cerrado, associated with Indigenous brigades and strategies to control late-season wildfires. Together, these trends highlight the adaptive, multifunctional, and regionally differentiated nature of Indigenous fire regimes in Brazil.

1. ***Agricultural fire***

Within the included corpus (n = 20), agricultural fire is the most consistently documented axis, appearing in 75% of the analyzed studies (n = 15; [10, 13, 20, 25, 26, 28, 32, 33, 35, 43, 52, 58, 64–66]). Our data extraction reveals that, across temporal phases, agricultural burning is described as intentional, cyclical, and socially coordinated, with a particular focus on the coivara systems of groups such as the Kayapó and Xavante. For instance, the analyzed records indicate that these burns are not merely for land clearing, but involve specific ‘resource island’ creation (apêtês) as detailed in studies from Amazonian and Cerrado transition zones. These descriptions are consistently documented across both early ethnographic accounts and recent governance-oriented studies included in this review, demonstrating a continuity of agricultural fire practices across the temporal phases of academic documentation.

To contextualize these findings within the broader literature, the coivara or swidden-fallow system represents the most widespread form of Indigenous agricultural fire management in tropical South America, documented since classical ethnographies in the Amazon and Cerrado [3, 19, 36, 58]. This rotational cycle involves selective clearing, controlled burning, and subsequent planting in soils enriched by ash rich in potassium, calcium, and magnesium [37, 55]. By alternating years of cultivation with ecological fallow, the system allows natural regeneration and the long-term maintenance of soil fertility, operating as a low-impact and adaptive form of land use [19, 43]. In the Cerrado, traditional burning cycles are typically concentrated at the onset of the dry season (March–May), aligning with ecological objectives such as stimulating fruiting and attracting fauna for subsistence hunting (Falleiro et al., 2020).

The specific case studies within our corpus (e.g., among the Kayapó, Paresi, Manoki, Myky, Macuxi, Munduruku, and Guarani peoples) demonstrate that fire serves multiple agricultural functions, including field clearing, weed control, and seed germination [23, 58]. In the Kayapó systems, it forms part of a broader ecological engineering strategy through the creation of “resource islands” (apêtês) and the enrichment of landscapes with useful species, resulting in complex “forest fields” that blend cultivation and forest structure [2, 4, 58]. Similarly, in the Cerrado, low-intensity burns accelerate nutrient cycling, promote herbaceous productivity, and reduce woody biomass accumulation, thus preventing large-scale fires and maintaining ecosystem stability [55].

Long-term archaeological and pedological evidence, such as the presence of Amazonian Dark Earths (ADEs), attests to the enduring influence of fire-based management on tropical soil fertility and landscape heterogeneity [34, 67]. Spatially, agricultural fire contributes to the formation of agroforestry mosaics that integrate phases of cultivation, regeneration, and mature forest, promoting structural diversity and sustaining biodiversity across temporal and spatial scales [4, 19, 23, 55].

Beyond its ecological and productive functions, agricultural fire also carries profound social and symbolic significance. Collective work, songs, and rituals performed during burning events transmit intergenerational knowledge and reinforce cultural identity and territorial belonging [23, 43]. In synthesis, agricultural fire exemplifies the reconciliation between production and conservation, challenging the persistent dichotomy between “agriculture and forest” and highlighting the coevolutionary relationship between Indigenous peoples and their environments [4, 23, 37].

2. ***Hunting and fruiting fire***

Hunting and fruiting fires are explicitly reported in 12 of the included studies [10, 25, 26, 28, 29, 33, 43, 47, 73], consistently associated with patch-mosaic dynamics and controlled early-season burns.

In the reviewed dataset, hunting and fruiting fire is disproportionately represented in Cerrado territories (Fig. 4), where mosaic-burning strategies are explicitly described in studies focusing on Xavante, Krahô, and Xerente territories. This biome-specific concentration supports the interpretation of fire as a regulatory ecological tool in savanna systems.

Among the Xavante, Krahô, Xerente, Wapichana, and other groups such as the Kayapó, Bororo, Paresi, and Macuxi, fire is strategically and collectively applied to attract fauna, stimulate fruiting, and control the accumulation of dry biomass [23, 43, 47, 74]. The intentional creation of mosaics composed of burned and unburned patches ensures a continuous supply of food resources and maintains habitat heterogeneity over time [38, 47]. Among the Krahô, the moral distinction between “good fire” (controlled and purposeful) and “bad fire” (uncontrolled and destructive) expresses a form of environmental ethics rooted in cosmology and practical knowledge [47, 74]. Similarly, Welch et al., [75] demonstrate that Xavante ritual and hunting fires contribute to savanna conservation by producing fine-scale spatial mosaics that enhance ecological resilience and resource diversity.

In the Xavante Cerrado, fire is integral to warã hunting expeditions, where collective coordination and ritualized burns are conducted at small scales to increase landscape heterogeneity and facilitate game tracking [74]. Among the Xerente (Akwẽ), the practice known as hàni mrore (“good fire”) is used to renew natural grasslands, stimulate the regrowth of edible and medicinal species, and safeguard villages against uncontrolled wildfires [76]. In the Amazonian savannas of Roraima (Lavrado), the Wapichana and Macuxi also apply fire at the beginning of the dry season to reduce fuel loads and maintain dynamic mosaics of grassland, secondary vegetation, and forest [5, 23].

From a broader ecological standpoint, hunting and fruiting fires operate as intermediate disturbances that regulate vegetation structure, sustain nutrient cycling, and enhance beta diversity across the landscape [9, 55]. In contrast, the data extracted from our review shows that Indigenous-managed fires are characterized by lower frequency, smaller extent, and reduced severity compared to accidental or agricultural burns, reflecting intentional control and ecological knowledge [11, 53]. Beyond their ecological functions, these practices hold deep social significance: collective burning and hunting reinforce reciprocity networks, food sovereignty, and community cohesion, embodying the integration of ecological stewardship with cultural continuity [47, 74].

3. ***Cultural and ritual fire***

Cultural and ritual uses of fire are documented in 9 of the included studies [10, 28, 33, 43, 47, 73], where fire is described not only as a productive tool but as a relational and cosmological agent embedded in territorial identity. These findings align with broader ethnographic theories where fire holds a central position in indigenous cosmologies as a living agent connecting humans, nature, and spirits [30, 43, 44, 71]. Among the Bororo, it integrates the Boe/Mári, linking burning with life and death transitions [16, 45]. Among the Xavante, it structures initiation (dahö’wa) and hunting rituals [73, 74]. Among the Krahô, the moral distinction between fires reinforces a relational ontology [21, 47]. In Chiriguano narratives, fire as a divine gift marks harvest and cleansing rituals [43, 69].

Ritual burns are spatially localized and low in frequency and intensity, contributing to biodiversity maintenance and fire regime modulation [11, 23, 53, 55]. Knowledge transmission involves natural signals (wind, humidity, phenology, constellations) and cosmological norms [23, 43].

4. ***Preventive and territorial defense fire***

Preventive and territorial defense burning emerges primarily in the most recent segment of the included corpus (2021–2025), appearing in studies such as Falleiro et al., [28], Oliveira et al., [53], Carvalho et al., [18], Fernandes et al., [32], Botelho et al., [10], and Espada et al., [25]. Our systematic analysis highlights that preventive fire has become a central governance tool across 95 Indigenous Territories, reflecting the institutionalization of Indigenous Fire Brigades and Integrated Fire Management (IFM) programs. Data extracted from these specific studies indicate that these practices resulted in a documented reduction of over 50% in catastrophic wildfire areas in territories such as the Kadiwéu, as reported by Oliveira et al., [53]. Through participatory planning, seasonal burn windows, and community-based monitoring, these brigades have become key actors in implementing IFM across diverse biomes, bridging traditional knowledge with technical fire control strategies.

In recent decades, Brazilian fire management policies have increasingly incorporated Indigenous knowledge into prevention strategies and Integrated Fire Management (IFM) programs, resulting in a hybrid model that bridges traditional and technical approaches [22, 23, 28]. The Federal Fire Brigades Program (Prevfogo/Ibama), created in 2013, stands as a central initiative within this framework, supporting Indigenous brigades among the Kadiwéu, Xerente, Kayapó, Pataxó, and Akrãtikatêjê peoples [8, 28]. These brigades combine local ecological calendars with monitoring technologies, enabling context-specific fire governance rooted in autonomy and intercultural collaboration.

Among the Kadiwéu, prescribed burns are used to form firebreaks, control invasive species, and renew grasslands, leading to a reduction of more than 50% in burned areas and a redistribution of fire seasons toward safer periods [53]. In the Xerente territory, seasonal planning integrates empirical knowledge with technical tools such as satellite imagery and weather forecasting, substantially reducing wildfire occurrence while strengthening local decision-making and territorial control [23, 76]. Similar arrangements among the Kayapó and Akrãtikatêjê peoples integrate agroforestry systems, ecological corridors, and territorial surveillance, reflecting a comprehensive approach to landscape management [23, 58, 74].

While our corpus focuses on the Brazilian context, these initiatives parallel adaptive co-management experiences developed in fire-prone regions such as northern Australia and the southwestern United States, which employ zoning systems, seasonal burning windows, and post-fire ecological assessments ([11]; Russell-Smith et al., 2012; [31]). Beyond ecological and preventive outcomes, the programs generate significant social benefits, including local empowerment, employment, technical training, and environmental education [28]. Within this context, Toledo and Macedo [70], conceptualize Integrated Fire Management as a lawful and adaptive governance strategy of the Pyrocene – a fire-dominated epoch – where traditional burning is recognized as a legitimate and essential component of Brazil’s emerging fire management paradigm.

5. ***Ecological and restorative fire***

Ecological and restorative fire management is documented in 10 included studies [10, 28, 33, 43, 53, 64, 65], where fire is framed as a controlled disturbance promoting regeneration, biodiversity maintenance, and fuel load reduction.

Within the reviewed corpus, ecological/restorative fire appears predominantly in Amazon and Pantanal territories (Table 1), indicating that restorative uses are especially documented in forested and wetland transition systems.

Consistent with the ecological principles identified in the wider literature [55, 67], the analyzed studies in our review employ fire as a tool for ecological restoration, used to control invasive grasses (Urochloa decumbens, Melinis minutiflora), stimulate native species regeneration, and reestablish functional clearings in savanna and forest landscapes [28, 53, 55, 67]. This approach reframes fire not as a destructive force, but as an ecological ally in restoring balance to degraded ecosystems.

Among the Kayapó, fire is integrated into agroforestry systems (sistemas agroflorestais, SAFs), where it stimulates the fruiting of culturally and economically significant species such as Caryocar brasiliense (pequi), Bertholletia excelsa (Brazil nut), and Bixa orellana (annatto) [4, 37, 58]. In Pataxó territories, strip burns are applied to favor native recolonization, reduce fire severity, and maintain ecological mosaics [23, 28]. Among the Kadiwéu, restorative fire management has been shown to reduce accidental burns and increase floristic diversity, while in Xacriabá lands, strategic burning helps control shrub encroachment, maintain gathering fields, and protect sacred areas [23, 47, 53].

Ecologically, these practices embody the principle of controlled disturbance, in which periodic, low-intensity fires enhance ecosystem resilience, sustain nutrient cycles, and prevent the spread of high-severity wildfires [9, 11, 55, 67]. Empirical studies reinforce this view: Pivello [56] demonstrated that controlled burns every one to two years are essential for maintaining Cerrado structure and avoiding catastrophic fires, while Falleiro et al., [29] showed that fire management centered on culturally significant species, such as pequi, promotes biodiversity recovery, native vegetation regeneration, and social engagement in restoration processes.

### Integration and overlapping uses

**Fig. 5:**
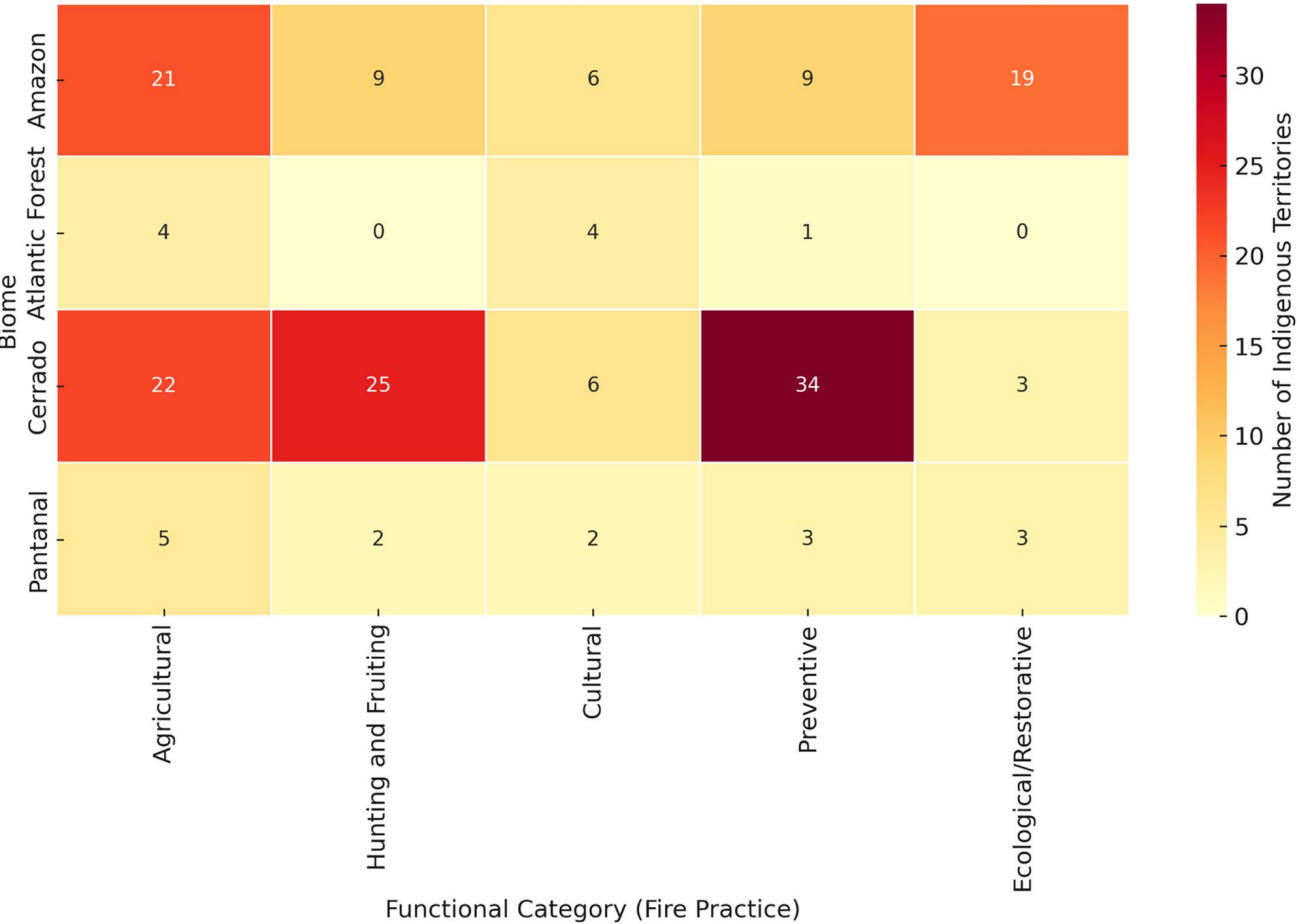
Heatmap showing the distribution of Indigenous Lands (ILs) according to the main functional fire categories across Brazilian biomes. The intensity of the color represents the number of Indigenous Territories associated with each fire practice within each biome. The five main fire-use categories are: Agricultural, Hunting and Fruiting, Cultural, Preventive, and Ecological/Restorative

The analysis of Table 2 and the biome heatmap (Fig. 5) derived from our systematic synthesis indicates that many Indigenous Lands documented in the corpus combine multiple fire functions within the same territorial context. This overlap is particularly evident in Cerrado territories, where agricultural, hunting, and preventive uses frequently co-occur. These patterns are derived directly from the classification of functional categories assigned during data extraction. To contextualize these findings, it is widely recognized that this functional rotation represents indigenous ecological engineering [4], creating successional mosaics and stable anthropogenic landscapes (“cultural forests”) maintained through controlled reburning [37, 53, 67]. Such integration enhances ecological resilience, food security, and reduces the risk of catastrophic fires [9, 23].

**Fig. 6:**
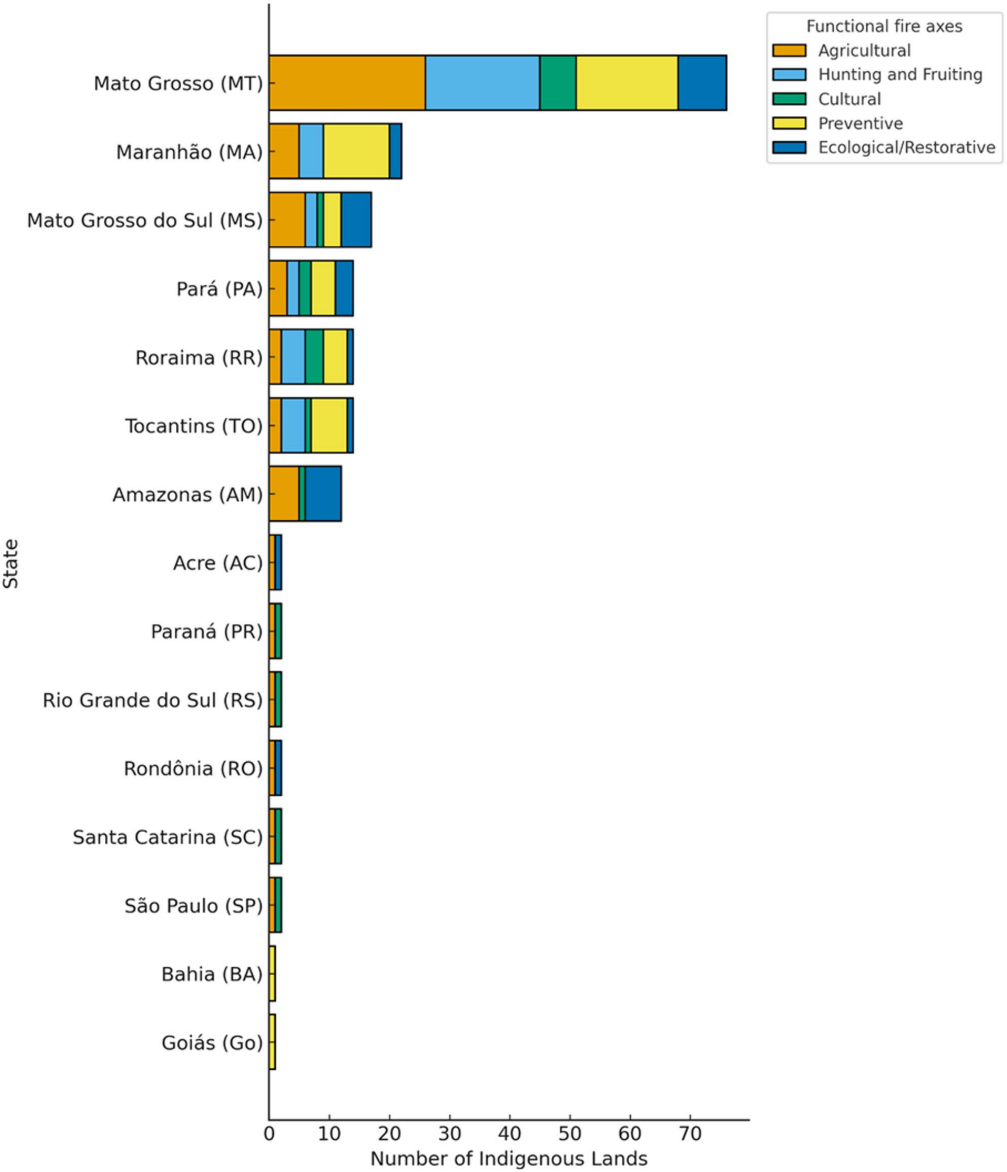
Fire management functions by state. Number of Indigenous lands per state grouped by functional fire axes

### Regional patterns and ecological implications

The synthesis of our reviewed corpus indicates that, in open biomes such as the Cerrado and Pantanal, fire functions as a regulatory tool for hunting, fruiting, and fuel load control. Our analysis of the included studies (e.g. [26, 47, 74]), confirms that controlled early-season burns are precisely aligned with ecological cycles and cultural calendars, preventing high-severity wildfires while maintaining productivity and habitat diversity. These findings are consistent with broader fire ecology theories, which characterize these regions as inherently pyric ecosystems where human-mediated disturbances play a regulatory role [9, 15, 55].

Specific evidence from the analyzed literature [26] documents that the reestablishment of traditional fire management in Indigenous Territories of western Mato Grosso restores ecological balance and reinforces traditional stewardship. Furthermore, data extracted from the review [53, 67, 74] show that the resulting successional mosaics enhance structural heterogeneity and limit the spatial spread of fire, promoting long-term resilience. Spatially, the distribution pattern identified in our study (Fig. 6) highlights a concentration of documented management in the Cerrado, particularly in Mato Grosso, Tocantins, and Mato Grosso do Sul, where agricultural and preventive uses predominate.

In contrast, in forested biomes such as the Amazon and the Atlantic Forest, fire assumes more restricted and agroecological roles. Its applications include soil fertilization, renewal of secondary succession, and land-use demarcation, occurring with low frequency and within specific humidity windows that minimize risk [4, 37, 55, 58]. These carefully timed burns sustain traditional agroforestry systems while avoiding the destructive effects associated with late dry-season fires.

From a landscape ecology perspective, Indigenous fire regimes increase spatiotemporal heterogeneity, sustain primary productivity, and mitigate the occurrence of severe wildfires [9, 11]. Empirical studies corroborate these patterns: Indigenous-managed landscapes exhibit lower recurrence and intensity of uncontrolled fires, alongside greater drought resilience and vegetation recovery capacity [53, 67]. Welch et al., [75] and Schmidt et al., [67] both demonstrate that Indigenous fire mosaics in fire-prone savannas enhance biodiversity and reduce the probability of catastrophic burns.

Collectively, these findings challenge technocratic “pyrophobia” and underscore that fire, when governed through socioecological principles, operates as a unifying element linking productive, ritual, and ecological dimensions of Indigenous life [23, 55, 74]. Indigenous fire management thus emerges not as a vestige of premodern practice, but as a dynamic, adaptive system central to contemporary strategies of conservation and climate resilience.

### Evidence beyond the reviewed corpus in underrepresented regions

While the 20 studies included in this review provide a robust cross-section of practices, certain regions did not yield eligible publications. To address these gaps, we consulted the broader anthropological and historical literature, which demonstrates that Indigenous fire management practices across multiple states and biomes, certain Brazilian regions did not yield eligible publications under the defined search strategy and inclusion criteria. Notably, portions of western Amazonia, semi-arid northeastern Brazil (Caatinga), and segments of the southern Atlantic Forest were either underrepresented or absent from the reviewed corpus.

This absence should not be interpreted as evidence of the absence of Indigenous fire use or management practices in those regions. Rather, it reflects the limitations of database-indexed, peer-reviewed publications that explicitly frame fire management as a central analytical focus. Many ethnographic, historical, or regionally published studies do not use standardized descriptors such as “fire governance” or “Integrated Fire Management,” which may limit their retrieval in systematic searches.

To address the geographical gaps identified in our systematic review, we draw upon broader anthropological and historical ecological literature demonstrates that Indigenous burning has long shaped landscapes across diverse portions of Amazonia, including western regions not represented in the reviewed corpus. As documented in this external research, historical ecology research documents Indigenous landscape engineering, agroforestry mosaics, and anthropogenic forests maintained through controlled burning practices [4, 19]. Evidence from Amazonian Dark Earths [34, 37] further supports the long-term role, even though these specific archaeological studies did not meet the inclusion criteria for our systematic analysis of contemporary management.

Similarly, ecological syntheses emphasize that fire plays a central role in tropical savannas globally and that human ignition practices have historically interacted with biome dynamics [9, 11]. In Brazil, Cerrado and transitional ecosystems beyond the specific territories represented in this review are widely recognized as shaped by low-intensity, seasonal burning regimes [55, 56], even where Indigenous governance is not explicitly framed in recent policy-oriented literature.

In northeastern Brazil, ethnobiological research conducted in Caatinga environments documents complex Traditional Ecological Knowledge systems that include fire-related land management, although these practices are often discussed under broader categories of resource management rather than fire governance per se (Alves & Albuquerque, 2010; Leal et al., 2005). The limited retrieval of Caatinga-based studies in this review therefore likely reflects differences in framing, terminology, and indexing rather than the absence of fire-related knowledge.

Taken together, these broader bodies of literature indicate that the spatial gaps identified in this review represent asymmetries in academic visibility and research infrastructure rather than the geographic distribution of Indigenous fire knowledge itself. The absence of eligible publications in certain states and biomes highlights the need for expanded collaborative research in underdocumented regions, particularly in western Amazonia and semi-arid northeastern Brazil, where Indigenous fire practices likely remain active but insufficiently represented in indexed scientific databases.

### Distribution of studies across indigenous ethnic groups

The 20 included studies collectively document fire management practices across 65 Indigenous ethnic groups and territorial contexts distributed across multiple Brazilian biomes, including the Amazon, Cerrado, Pantanal, and Atlantic Forest.; however, the distribution of research is markedly uneven. Data extracted from our review shows a significant concentration on groups such as the Kayapó [23, 58, 66], Xavante [47, 73, 74], Xerente [18, 76], Krahô [47], Paresi and Kadiwéu [28, 53]. These groups are predominantly located in the Cerrado and transitional forest–savanna regions. This asymmetry highlights a key finding of our study: scientific production currently reflects the geography of research infrastructure rather than the full diversity of Indigenous knowledge.

In contrast, numerous Indigenous ethnic groups, especially in western Amazonia and the southern Atlantic Forest, remain underrepresented or entirely absent from indexed academic literature on fire management. This asymmetry suggests that current scientific production reflects the geography of research infrastructure and policy-driven fire management initiatives rather than the full diversity of Indigenous fire knowledge systems in Brazil.

Preliminary comparisons indicate both shared and context-specific features across groups. Early dry-season mosaic burning is widely documented among Cerrado peoples such as the Xavante and Krahô [47, 74], whereas agroforestry-oriented and restorative uses are more frequently described among Amazonian groups such as the Kayapó [58, 67]. These patterns suggest that variation in fire management reflects both ecological context and culturally embedded governance systems.

Considering that Brazil is home to more than 300 Indigenous ethnic groups, the reviewed corpus documents fire practices in a relatively small fraction of them, reinforcing the need for expanded collaborative and intercultural research initiatives.

### Research concentration and structural asymmetries

The included corpus reveals a marked concentration of territorial data within a limited number of research groups. Five recent multi-territory studies [10, 25, 28, 29, 32] account for a substantial proportion of documented Indigenous Lands. Rather than reflecting methodological bias, this concentration indicates structural asymmetries in research funding, institutional capacity, and long-term engagement in fire-prone regions such as Mato Grosso, Tocantins, and Maranhão.

This pattern suggests that the apparent territorial clustering in the review mirrors the geography of scientific production as much as the geography of Indigenous fire practice itself. The uneven distribution of research underscores the need for expanded collaborative studies in underdocumented regions, particularly in western Amazonia.

### Interpretation of research concentration patterns

The concentration of studies in specific states, particularly Mato Grosso, Tocantins, and Maranhão, and in biomes such as the Cerrado and transitional forest–savanna systems reflects a combination of historical and institutional factors rather than a simple gradient of academic “interest.”

Two partially overlapping dynamics can be identified within the reviewed corpus. First, early ethnographic and ecological studies (e.g. [33, 43, 58]), were conducted independently by different researchers working in territories that later became reference cases in fire ecology debates. This produced a historical accumulation of literature in certain regions, especially in Amazon–Cerrado transition zones.

Second, more recent concentration patterns are strongly associated with coordinated research programs linked to Integrated Fire Management (IFM) and Indigenous Fire Brigades (e.g. [10, 25, 28, 32, 53]), . These initiatives involve multi-territory monitoring projects supported by federal institutions such as Prevfogo/Ibama, resulting in clusters of publications emerging from shared research networks.

Therefore, the spatial concentration identified in this review reflects both cumulative historical scholarship and contemporary institutionalized research efforts. It does not necessarily indicate greater Indigenous fire activity in those territories, but rather greater academic visibility and sustained research infrastructure in specific regions.

### Governance and public policy

Our systematic review identifies a clear temporal shift in the documented literature, indicating that traditional fire management practices have been increasingly incorporated into Brazilian environmental policies, marking a paradigm shift from the “zero-fire” approach toward adaptive co-management frameworks, a move widely discussed in broader policy literature [22, 23]. Specifically, the studies included in our corpus (e.g., Falleiro et al., [28], Oliveira [53], document how the *Prevfogo/Ibama* program, established in 2013, catalyzed this transformation by creating Indigenous Fire Brigades among the Kadiwéu, Xerente, Pataxó, Kayapó, and Akrãtikatêjê peoples. The analysis of these selected publications reveals that, through participatory planning, seasonal burn windows, and community-based monitoring, these brigades have become key actors in implementing Integrated Fire Management (IFM) across diverse biomes [8, 53, 76].

Empirical assessments confirm the ecological and social effectiveness of these initiatives. Lazzarini et al., [42] documented significant reductions in wildfire frequency and strengthened local governance capacity across the Xerente, Kraolândia, Funil, and Araguaia territories. Carvalho et al., [18] further highlighted the positive perception of fire use among 28 Xerente villages in Tocantins, where respondents associated IFM training with improvements in both food security and environmental stewardship. In turn, Falleiro et al., [29] demonstrated how participatory monitoring centered on the cultural keystone species *Caryocar brasiliense* (pequi) supports ecological recovery and reinforces Indigenous-led governance in Paresi, Kadiwéu, and Xerente lands. Complementarily, satellite analyses of Kadiwéu territory revealed over 50% reductions in burned area and associated carbon emissions [53, 67].

Brazilian IFM arrangements now align with global Indigenous Fire Stewardship (IFS) models developed in regions such as northern Australia, emphasizing Indigenous leadership and co-management as central principles (Russell-Smith et al., 2012; [22, 23]). Building on this framework, Sautchuk et al., [63] conceptualize fire governance as a sociotechnical system co-produced by Indigenous communities, state agencies, and scientific institutions through joint processes of monitoring, training, and policymaking. Similarly, Bilbao et al., [8] advocate participatory and intercultural governance structures across South America that foreground Indigenous leadership in decision-making and management. Nonetheless, Miguel [46], warns that policy rollbacks and weak institutional enforcement threaten these advances, linking recent increases in fire incidence to socio-political instability in the Brazilian Amazon.

### Sociocultural and epistemological implications

The synthesis of the reviewed corpus suggests that Indigenous fire management embodies a sophisticated system of Traditional Ecological Knowledge (TEK) grounded in observation, orality, sensory experience, and ritualization [43]. When interpreted through broader theoretical frameworks, these practices resonate with established concepts of TEK [6] and anthropological insights into relational ontologies [71]. Specifically, findings extracted from the analyzed studies [23, 47] indicate that fire is treated not merely as a tool but as an agentive entity that demands respect, timing, and responsibility. This relational perspective aligns with wider scholarship on Amerindian perspectivism [71] and other non-dualist cosmologies in which humans, nonhumans, and ecological processes are intertwined in networks of reciprocity [21]. Furthermore, the evidence from our review supports the view that Indigenous fire management operates as a form of local science, following iterative observation–testing–correction cycles comparable to empirical methods in Western science [1, 6, 40], , yet embedded within distinct epistemological frameworks. This is exemplified by recent publications within our corpus [63], which describe these hybrid systems as *sociotechnical assemblages* where traditional expertise and environmental technologies coevolve through intercultural governance.

Historically, pyrophobic conservation policies criminalized these practices and marginalized the knowledge systems behind them [43, 55]. However, mounting evidence demonstrates that total fire suppression leads to fuel accumulation, ecosystem degradation, and catastrophic wildfires [11, 61]. The recognition of the preventive and restorative functions of Indigenous burning reframes the debate, transforming fire from a perceived “risk” into a form of *relationship*, a process of care, reciprocity, and ecological regulation [23, 28, 53].

Recent socioecological studies further illustrate this shift. Santopuoli et al., [62], examining community perceptions in the Tocantins Cerrado, found that most wildfires are anthropogenic in origin and that local awareness and environmental education substantially improve mitigation practices. In this context, the act of conserving is inseparable from the act of burning wisely, a principle that encapsulates the biocultural essence of Indigenous fire governance [55, 74].

Nevertheless, considering that Brazil is home to more than 300 Indigenous ethnic groups, our bibliometric review revealed that studies addressing Indigenous fire management have been conducted in less than 15% of these groups, underscoring the vast body of knowledge yet to be co-produced with Indigenous peoples. These studies are spatially concentrated in states such as Mato Grosso, Tocantins, and Maranhão, regions characterized by both intense fire regimes and high cultural and territorial diversity. In contrast, significant knowledge gaps persist in vast areas of the western Amazon, where Indigenous burning practices remain poorly documented or entirely absent from academic literature.

This lack of documentation gains particular urgency in the context of climate change, as Indigenous and non-Indigenous communities alike are becoming increasingly vulnerable to extreme fire events that surpass historical experience. Addressing these challenges demands the co-production of knowledge and adaptive fire strategies across broader territorial and institutional scales. Importantly, this gap reflects not the absence of Indigenous fire knowledge, but the limited documentation of such knowledge within indexed academic literature.

### Study limitations

Despite the rigor of the PRISMA guidelines, this systematic review has limitations that should be considered. First, the reliance on indexed, peer-reviewed databases resulted in a relatively small corpus (*n* = 20), which likely excludes a significant volume of information contained in gray literature, technical reports, and non-indexed ethnographic accounts. Consequently, the 95 Indigenous Territories documented here represent only a fraction of the territories where fire management is actively practiced in Brazil. Second, there is a marked geographic and thematic bias; research is heavily concentrated in fire-prone savannas (Cerrado) and major conservation frontiers, leaving biomes like the Caatinga and Southern Atlantic Forest underrepresented. Finally, the focus on contemporary management practices meant that relevant archaeological and long-term historical ecology studies were excluded from the formal systematic count, although they were used to contextualize the discussion. These gaps underscore the need for expanded research infrastructure and the inclusion of diverse knowledge formats in future assessments of Indigenous fire governance.

## Conclusions

The integrated analysis of the 20 peer-reviewed studies included in this systematic review indicates that, within the scope of the documented academic literature, Indigenous fire management in Brazil is consistently described as a multifunctional, adaptive, and territorially embedded system that interweaves ecological, social, cultural, and political dimensions. Rather than being framed solely as a technical land-use practice, the reviewed studies portray fire management as part of broader relational and cosmological frameworks that integrate empirical environmental observation with social organization and cultural continuity.

Across the biomes represented in the reviewed corpus, Indigenous fire practices are documented as context-dependent strategies shaped by local ecological conditions and seasonal cycles. In open environments such as the Cerrado and Pantanal, several studies describe early dry-season burning as a mechanism for regulating fuel loads, maintaining vegetation structure, and reducing the likelihood of high-severity wildfires. In forested regions, the included literature reports the use of controlled burns for soil fertilization, agroforestry management, and the renewal of secondary vegetation. These findings suggest that, within the documented cases, fire operates as a regulated disturbance that contributes to landscape heterogeneity and ecological resilience.

From a sociocultural perspective, the reviewed studies consistently emphasize that fire is embedded in systems of knowledge transmission, ritual practice, and territorial governance. However, it is important to stress that these interpretations are derived from the specific Indigenous groups and territories represented in the included studies and should not be generalized to all Indigenous peoples in Brazil. The corpus documents a subset of ethnic groups and regions, with clear spatial concentration in the Cerrado and parts of the eastern Amazon.

Politically, the literature reviewed here highlights a documented shift in certain regions toward the incorporation of Indigenous fire practices into Integrated Fire Management (IFM) and co-management frameworks. These cases illustrate emerging forms of intercultural governance in which Indigenous knowledge and institutional fire management strategies interact. Nonetheless, the extent to which such arrangements are representative across Brazil remains uncertain and warrants further investigation.

It is also important to recognize that global warming and climate change may increasingly affect Indigenous groups’ ability to use and manage fire. Alterations in rainfall regimes, temperature patterns, and extreme weather events can modify ecosystem dynamics, fuel accumulation, and fire behavior, while simultaneously disrupting the climatic cues, seasonal indicators, and plant phenological cycles that underpin traditional fire calendars. Such transformations may challenge the temporal and ecological foundations upon which Indigenous fire management systems rely, requiring adaptive responses and renewed intercultural dialogue in fire governance.

Taken together, the reviewed evidence suggests that Indigenous fire management, as documented in peer-reviewed literature, offers important insights for discussions on biodiversity conservation, wildfire prevention, and adaptive governance. At the same time, the limited territorial and epistemological coverage of the available academic record underscores the need for expanded research efforts, particularly in underdocumented regions and among Indigenous groups not represented in the current corpus.

Accordingly, the conclusions presented here should be interpreted as a synthesis of documented patterns and research trends within indexed scientific production, rather than as an exhaustive representation of Indigenous fire knowledge and practice across Brazil. Future research that engages broader territorial coverage, climate-related transformations, and diverse documentation sources will be essential to deepen and refine this understanding.

**Table 2:** Brazilian Indigenous Lands (ILs) documented with fire management practices, showing the state, biome, functional category, and corresponding bibliographic references

Indigenous Lands (IL)	State	Biome	Functional category	Reference
Xavante	Mato Grosso (MT)	Cerrado	Hunting and Fruiting	Welch [73],
Kayapó	Pará (PA)	Amazon	Hunting and Fruiting + Ecological/Restorative	Posey [58],
Krahô	Tocantins (TO)	Cerrado	Agricultural + Hunting and Fruiting	Mistry et al., [47]
Irantxe	Mato Grosso (MT)	Cerrado	Agricultural + Hunting and Fruiting	Falleiro. [26]
Myky	Mato Grosso (MT)	Cerrado	Agricultural + Hunting and Fruiting	Falleiro. [26]
Paresí	Mato Grosso (MT)	Cerrado	Agricultural + Hunting and Fruiting	Falleiro. [26]
Tirecatinga	Mato Grosso (MT)	Cerrado	Agricultural + Hunting and Fruiting	Falleiro. [26]
Utiariti	Mato Grosso (MT)	Cerrado	Agricultural + Hunting and Fruiting	Falleiro. [26]
Kuikuro	Mato Grosso (MT)	Amazon	Agricultural + Ecological/Restorative	Carneiro [13],
Munduruku	Amazonas (AM)	Amazon	Ecological/Restorative	Frikel [33],
Tikuna	Amazonas (AM)	Amazon	Agricultural + Ecological/Restorative	Hammond et al., [35]
Tuyuka	Amazonas (AM)	Amazon	Agricultural + Ecological/Restorative	Schmidt [65],
Yanomami	Roraima (RR)	Amazon	Hunting and Fruiting + Ecological/Restorative	Frikel [33],
Aruak	Amazonas (AM)	Amazon	Ecological/Restorative	Frikel [33],
Maku	Acre (AC)	Amazon	Agricultural + Ecological/Restorative	Frikel [33],
Kadiwéu	Mato Grosso do Sul (MS)	Pantanal	Agricultural + Ecological/Restorative	Frikel [33],
Canela	Maranhão (MA)	Cerrado	Agricultural + Ecological/Restorative	Frikel [33],
Povos do Alto Xingu	Mato Grosso (MT)	Amazon	Agricultural + Hunting and Fruiting + Ecological/Restorative	Frikel [33],
Kawaiwete	Mato Grosso (MT)	Amazon	Agricultural	Denevan [20],
Kokama	Amazonas (AM)	Amazon	Agricultural	Noda et al., [52]
Kuikuro	Mato Grosso (MT)	Amazon	Agricultural	Schmidt and Heckenberger [66],
Pataxó	Bahia (BA)	Atlantic Forest	Prevention and Territorial Defense	Falleiro et al., [28]
Macuxi	Roraima (RR)	Amazon	Hunting and Fruiting + Cultural/Ritual + Prevention and Territorial Defense	Falleiro et al., [28]
Taurepang	Roraima (RR)	Amazon	Agricultural + Hunting and Fruiting + Prevention and Territorial Defense	Falleiro et al., [28]
Povos do Lavrado	Mato Grosso (MT)	Cerrado	Agricultural + Prevention and Territorial Defense	Falleiro et al., [28]
Povos da Bacia do Xingu	Mato Grosso (MT)	Cerrado	Agricultural + Prevention and Territorial Defense	Falleiro et al., [28]
Povos da Bacia do Araguaia	Pará (PA)	Cerrado	Agricultural + Prevention and Territorial Defense	Falleiro et al., [28]
Kadiwéu	Mato Grosso (MT)	Cerrado	Agricultural + Prevention and Territorial Defense	Falleiro et al., [28]
Xavante	Mato Grosso (MT)	Cerrado	Agricultural + Hunting and Fruiting + Prevention and Territorial Defense	Falleiro et al., [28]
Kayapó	Pará (PA)	Amazon	Agricultural + Prevention and Territorial Defense + Ecological/Restorative	Falleiro et al., [28]
Juruna	Mato Grosso (MT)	Amazon	Agricultural + Hunting and Fruiting + Prevention and Territorial Defense	Falleiro et al., [28]
Nambikwara	Mato Grosso (MT)	Cerrado	Agricultural + Hunting and Fruiting + Prevention and Territorial Defense	Falleiro et al., [28]
Paresi	Mato Grosso (MT)	Cerrado	Agricultural + Prevention and Territorial Defense	Falleiro et al., [28]
Manoki (Irantxe)	Mato Grosso (MT)	Cerrado	Agricultural + Hunting and Fruiting + Cultural/Ritual + Prevention and Territorial Defense	Falleiro et al., [28]
Myky	Mato Grosso (MT)	Cerrado	Agricultural + Hunting and Fruiting + Prevention and Territorial Defense	Falleiro et al., [28]
Kawaieté	Mato Grosso (MT)	Amazon	Prevention and Territorial Defense	Falleiro et al., [28]
Enawenê-Nawê	Mato Grosso (MT)	Cerrado	Agricultural + Prevention and Territorial Defense	Falleiro et al., [28]
Krahô	Tocantins (TO)	Cerrado	Cultural/Ritual + Prevention and Territorial Defense	Falleiro et al., [28]
Wapichana	Roraima (RR)	Amazon	Agricultural + Hunting and Fruiting + Cultural/Ritual + Prevention and Territorial Defense	Falleiro et al., [28]
Akrãtikatêjê	Pará (PA)	Amazon	Prevention and Territorial Defense	Falleiro et al., [28]
Rikbaktsa	Mato Grosso (MT)	Cerrado	Cultural/Ritual + Prevention and Territorial Defense	Falleiro et al., [28]
Suruí	Pará (PA)	Amazon	Cultural/Ritual + Prevention and Territorial Defense	Falleiro et al., [28]
Karitana	Roraima (RR)	Amazon	Cultural/Ritual + Prevention and Territorial Defense	Falleiro et al., [28]
Xavante	Mato Grosso (MT)	Cerrado	Hunting and Fruiting + Cultural/Ritual + Ecological/Restorative	Welch et al., [74]
Kayapó	Pará (PA)	Amazon	Agricultural + Hunting and Fruiting + Cultural/Ritual + Ecological/Restorative	Leonel [43],
Nambikwara	Mato Grosso (MT)	Cerrado	Hunting and Fruiting + Prevention and Territorial Defense	Leonel [43],
Xavante	Mato Grosso (MT)	Cerrado	Hunting and Fruiting + Cultural/Ritual	Leonel [43],
Avá-Guarani	São Paulo (SP)	Atlantic Forest	Agricultural + Cultural/Ritual	Leonel [43],
Avá-Guarani	Paraná (PR)	Atlantic Forest	Agricultural + Cultural/Ritual	Leonel [43],
Avá-Guarani	Santa Catarina (SC)	Atlantic Forest	Agricultural + Cultural/Ritual	Leonel [43],
Avá-Guarani	Rio Grande do Sul (RS)	Atlantic Forest	Agricultural + Cultural/Ritual	Leonel [43],
Desana	Amazonas (AM)	Amazon	Agricultural + Cultural/Ritual + Ecological/Restorative	Leonel [43],
Munduruku	Amazonas (AM)	Amazon	Agricultural + Ecological/Restorative	Leonel [43],
Kamaiurá	Mato Grosso (MT)	Amazon	Agricultural + Ecological/Restorative	Leonel [43],
Bororo	Mato Grosso (MT)	Cerrado	Cultural/Ritual	Leonel [43],
Suruí	Rondônia (RO)	Amazon	Agricultural + Ecological/Restorative	Leonel [43],
Guajá	Maranhão (MA)	Amazon	Agricultural + Ecological/Restorative	Leonel [43],
Kadiwéu	Mato Grosso do Sul (MS)	Pantanal	Prevention and Territorial Defense + Ecological/Restorative	Oliveira et al., [53]
Xerente	Tocantins (TO)	Cerrado	Agricultural + Prevention and Territorial Defense	Carvalho et al., [18]
Bakairi	Mato Grosso (MT)	Cerrado	Hunting and Fruiting	Falleiro et al., [29]
Xerente	Tocantins (TO)	Cerrado	Hunting and Fruiting + Prevention and Territorial Defense	Falleiro et al., [29]
Krahô	Tocantins (TO)	Cerrado	Hunting and Fruiting + Prevention and Territorial Defense	Falleiro et al., [29]
Paresi	Mato Grosso (MT)	Cerrado	Hunting and Fruiting + Prevention and Territorial Defense	Falleiro et al., [29]
Gavião	Maranhão (MA)	Cerrado	Hunting and Fruiting + Prevention and Territorial Defense	Falleiro et al., [29]
Guajajara	Maranhão (MA)	Cerrado	Hunting and Fruiting + Prevention and Territorial Defense	Falleiro et al., [29]
Krikati	Maranhão (MA)	Cerrado	Hunting and Fruiting	Falleiro et al., [29]
Kanela	Maranhão (MA)	Cerrado	Prevention and Territorial Defense	Falleiro et al., [29]
Alto Xingu	Mato Grosso (MT)	Cerrado	Prevention and Territorial Defense	Falleiro et al., [29]
Araguaia	Tocantins (TO)	Cerrado	Prevention and Territorial Defense	Falleiro et al., [29]
Avá-Canoeiro	Goiás (Go)	Cerrado	Prevention and Territorial Defense	Falleiro et al., [29]
Juininha	Mato Grosso (MT)	Cerrado	Hunting and Fruiting + Prevention and Territorial Defense	Falleiro et al., [29]
Utiariti	Mato Grosso (MT)	Cerrado	Prevention and Territorial Defense	Falleiro et al., [29]
Apinajé	Tocantins (TO)	Cerrado	Hunting and Fruiting	Falleiro et al., [29]
Porquinhos	Maranhão (MA)	Cerrado	Hunting and Fruiting	Falleiro et al., [29]
Governador/Krikati	Maranhão (MA)	Cerrado	Prevention and Territorial Defense	Falleiro et al., [29]
Araribóia	Maranhão (MA)	Cerrado	Prevention and Territorial Defense	Falleiro et al., [29]
Guajajara	Maranhão (MA)	Cerrado	Agricultural + Prevention and Territorial Defense	Fernandes et al., [32]
Awá-Guajá	Maranhão (MA)	Cerrado	Agricultural + Prevention and Territorial Defense	Fernandes et al., [32]
Gavião Pykopjê	Maranhão (MA)	Cerrado	Prevention and Territorial Defense	Fernandes et al., [32]
Tabajara	Maranhão (MA)	Cerrado	Prevention and Territorial Defense	Fernandes et al., [32]
Krikati	Maranhão (MA)	Cerrado	Agricultural + Prevention and Territorial Defense	Fernandes et al., [32]
Xerente and Funil	Tocantins (TO)	Cerrado	Prevention and Territorial Defense + Ecological/Restorative	Espada et al., [25]
Gavião	Maranhão (MA)	Cerrado	Prevention and Territorial Defense	Espada et al., [25]
Bororo	Mato Grosso (MT)	Pantanal	Cultural/Ritual + Agricultural	Botelho et al., [10]
Guató	Mato Grosso (MT)	Pantanal	Hunting and Fruiting + Agricultural	Botelho et al., [10]
Guató	Mato Grosso do Sul (MS)	Pantanal	Hunting and Fruiting + Agricultural	Botelho et al., [10]
Terena	Mato Grosso do Sul (MS)	Pantanal	Agricultural + Prevention and Territorial Defense	Botelho et al., [10]
Kadiwéu	Mato Grosso do Sul (MS)	Pantanal	Cultural/Ritual + Prevention and Territorial Defense	Botelho et al., [10]
Guarani-Kaiowá/Chamacoco	Mato Grosso do Sul (MS)	Pantanal	Hunting and Fruiting	Botelho et al., [10]
Macro-Jê	Mato Grosso (MT)	Pantanal	Agricultural + Ecological/Restorative	Botelho et al., [10]
Macro-Jê	Mato Grosso do Sul (MS)	Pantanal	Agricultural + Ecological/Restorative	Botelho et al., [10]
Aruak	Mato Grosso (MT)	Pantanal	Agricultural + Ecological/Restorative	Botelho et al., [10]
Aruak	Mato Grosso do Sul (MS)	Pantanal	Agricultural + Ecological/Restorative	Botelho et al., [10]
Xaraés	Mato Grosso (MT)	Pantanal	Agricultural + Ecological/Restorative	Botelho et al., [10]
Xaraés	Mato Grosso do Sul (MS)	Pantanal	Agricultural + Ecological/Restorative	Botelho et al., [10]

Functional categories refer to the main uses and meanings attributed to fire, agricultural, hunting and fruiting, cultural, preventive, or ecological

## Data Availability

All data analyzed in this study are derived from publicly available sources, as cited in the manuscript. The complete dataset supporting the findings (including PRISMA flowchart, reference list, and summarized table of studies) is provided in the Supplementary Materials.

## References

[1] Agrawal A. Dismantling the divide between indigenous and scientific knowledge. Dev Change. 1995;26(3):413–39. 10.1111/j.1467-7660.1995.tb00560.x.

[2] Anderson AB, Posey DA. Management of a tropical scrub savanna by the Gorotire Kayapó of Brazil. Adv Econ Bot. 1989;7:159–73.

[3] Balée W. Footprints of the forest: Ka’apor ethnobotany—the historical ecology of plant utilization by an Amazonian people. New York: Columbia University; 1994.

[4] Balée W. The research program of historical ecology. Annu Rev Anthropol. 2006;35:75–98.10.1146/annurev.anthro.35.081705.123231 .

[5] Barbosa RI, Fearnside PM. Fire frequency and area burned in the Roraima savannas of northern Brazil. J Trop Ecol. 2005;21(1):25–33.10.1016/j.foreco.2004.09.011.

[6] Berkes F. Sacred ecology. 3rd ed. London: Routledge; 2012.

[7] Bilbao BA, Leal AV, Méndez CL. Indigenous use of fire and forest loss in Canaima National Park, Venezuela: Assessment of and tools for alternative strategies of fire management in Pemón indigenous lands. Hum Ecol. 2010;38(4):663–73. oi.org/10.1007/s10745-010-9344-0.

[8] Bilbao BA, Mistry J, Millán A, Berardi A, Rodríguez A. Sharing multiple perspectives on burning: Towards a participatory and intercultural fire management policy in Venezuela, Brazil, and Guyana. Fire. 2019;2(3):39. 10.3390/fire2030039.

[9] Bond WJ, Keeley JE. Fire as a global herbivore: The ecology and evolution of flammable ecosystems. Trends Ecol Evol. 2005;20(7):387–94. 10.1016/j.tree.2005.04.025. 16701401 10.1016/j.tree.2005.04.025

[10] Botelho MT, de Arruda, Chiaravalloti RM, Berlinck CN. Playing with fire: The vital influence of traditional knowledge on the socio-biodiversity of the Pantanal. Biodivers Bras. 2024;14(4):155–68.10.37002/biodiversidadebrasileira.v14i4.2547.

[11] Bowman DMJS, Balch J, Artaxo P, Bond WJ, Carlson JM, Cochrane MA, D’Antonio CM, DeFries RS, Doyle JC, Harrison SP, Johnston FH, Keeley JE, Krawchuk MA, Kull CA, Marston JB, Moritz MA, Prentice IC, Roos CI, Scott AC, et al. The human dimension of fire regimes on Earth. J Biogeogr. 2011;38(12):2223–36. 10.1111/j.1365-2699.2011.02595.x. 22279247 10.1111/j.1365-2699.2011.02595.x PMC3263421

[12] Bramer WM, Rethlefsen ML, Kleijnen J, Franco OH. Optimal database combinations for literature searches in systematic reviews: A prospective exploratory study. Syst Rev. 2017;6(1):245. 10.1186/s13643-017-0644-y. 29208034 10.1186/s13643-017-0644-y PMC5718002

[13] Carneiro RL. The cultivation of manioc among the Kuikuru of the Upper Xingu. In: Hames RB, Vickers WT, editors. Adaptive responses of native Amazonians. Cambridge (MA): Academic; 1983. pp. 65–111.

[14] Costa AB, Zoltowski APC. Como escrever um artigo de revisão sistemática. In: Koller SH, Couto MCPP, Hohendorff JV, editors. Manual de produção científica. Porto Alegre: Editora Penso; 2014. pp. 53–67.

[15] Coutinho LM. Fire in the ecology of the Brazilian cerrado. Ecol Mediterr. 1990;16(1–2):85–94.

[16] Crocker JC. Vital souls: Bororo cosmology, natural symbolism, and shamanism. Tucson (AZ): University of Arizona; 1985.

[17] Dantas C, Medeiros Costa Neto E, Azevedo Koch EB. Understanding fire through ethnoecology in Brazil. Ethnobiol Lett. 2025;16(1):1–37. 10.14237/ebl.16.1.2025.1942.

[18] de Carvalho EV, Oliveira LM, Cachoeira JN, Silva ADP, Santos AF. Fogo no Cerrado em terras indígenas Xerente (Tocantins): pesquisa de opinião em comunidade indígena. Bol Mus Para Emílio Goeldi Cienc Hum. 2023;18(2): 1-10 . 10.46357/bcnaturais.v18i2.935

[19] Denevan WM. Cultivated landscapes of native Amazonia and the Andes: Triumph over the soil. Oxford: Oxford University Press; 2001.

[20] Denevan WM. Semi-intensive pre-European cultivation and the origins of anthropogenic dark earths in Amazonia. In: Glaser B, Woods WI, editors. Amazonian dark earths: Explorations in space and time. Berlin: Springer; 2004. pp. 135–43.

[21] Descola P. *Beyond nature and culture.* Chicago (IL). University of Chicago Press; 2013. 10.7208/chicago/9780226145006.001.0001.

[22] Eloy L, Hecht S, Steward A, Mistry J. Firing up: Policy, politics and polemics under new and old burning regimes. Geogr J. 2019;185(1):2–9. 10.1111/geoj.12293.

[23] Eloy L, Falleiro RM, Silva EM, Schmidt IB. Manejo do fogo por povos indígenas e comunidades tradicionais no Brasil. In: Eloy L, Falleiro RM, Schmidt IB, Oliveira MR, editors. *Manejo integrado do fogo em terras indígenas brasileiras: Povos indígenas e comunidades tradicionais e o manejo do fogo.* Brasília: Instituto Brasileiro do Meio Ambiente e dos Recursos Naturais Renováveis – IBAMA/Prevfogo; 2021. pp. 94–115. Available from: https://hal.science/hal-0321151

[24] Eriksen C, Hankins DL. The retention, revival, and subjugation of Indigenous fire knowledge through agency fire fighting in eastern Australia and California. Soc Nat Resour. 2014;27(12):1288–303. oi.org/10.1080/08941920.2014.918226.

[25] Espada ALV, Oliveira MS, d’Assumpção M, Xerente PP, Xerente D, Xerente AS. Breaking barriers in the Brazilian Amazon: Indigenous women’s roles in climate action and fire management. In: Rogelj T, Kroese L, editors. *Women as stewards of forests. Trop For Issues.* 2025;63:124–31. Wageningen: Tropenbos International; 2025. 10.55515/OWBQ7111.

[26] Falleiro RM. Resgate do manejo tradicional do Cerrado com fogo para proteção das terras indígenas do oeste do Mato Grosso: um estudo de caso. Biodivers Bras. 2011;1(2):86–96. 10.37002/biodiversidadebrasileira.v1i2.114.

[27] Falleiro RM, Santana MT, Berni CR. As contribuições do Manejo Integrado do Fogo para o controle dos incêndios florestais nas Terras Indígenas do Brasil. Biodivers Bras. 2016;6(2):88–105. 10.37002/biodiversidadebrasileira.v6i2.655.

[28] Falleiro RM, Steil L, Oliveira MS, Lando I, Machado LOR, Cunha AMC, Zacharias GC. Histórico, avaliação, oportunidades e desafios do manejo integrado do fogo nas terras indígenas brasileiras. Biodivers Bras. 2021;11(2):116–51. 10.37002/biodiversidadebrasileira.v11i2.1742.

[29] Falleiro RM, Moura LC, Xerente PP, Pinto CP, Santana MT, Corrêa MA, Schmidt IB. Using a cultural keystone species in participatory monitoring of fire management in Indigenous Lands in the Brazilian savanna. Fire. 2024;7(7):231. 10.3390/fire7070231.

[30] Fausto C. Donos demais: Maestria e domínio na Amazônia. Mana. 2008;14(2):329–66. 10.1590/S0104-93132008000200003.

[31] Fernandes PM, Davies G, Ascoli D, Fernández C, Moreira F, Rigolot E, Stoof CR, Vega JA, Molina D. Prescribed burning in southern Europe: Developing fire management in a dynamic landscape. Front Ecol Environ. 2013;11(Suppl 1):e4–14. 10.1890/120298.

[32] Fernandes HGP, Costa WS, Nogueira FLS, Braga EV, Leão PHA, Rodrigues TCS, Silva Júnior CHL. Análise do uso e cobertura da terra e suas relações com o fogo nas Terras Indígenas do município de Amarante, Maranhão, Brasil. Revista Brasileira de Geografia Física. 2024;17(3):1738–53. ttps://doi.org/10.26848/rbgf.v17.3.p1738-1753.

[33] Frikel P. Arboricultura e agricultura indígena: considerações etnológicas e arqueológicas sobre as culturas do fogo na Amazônia. *Bol Mus Para Emílio Goeldi Ser Antropol.* 1978;68:1–20. Available from: http://www.etnolinguistica.org/biblio:frikel-1978-arboricultura

[34] Glaser B, Woods WI. Amazonian dark earths: Explorations in space and time. Berlin: Springer-; 2004. 10.1007/978-3-662-05683-7.

[35] Hammond DS, Dolman PM, Watkinson AR. Modern Ticuna swidden-fallow management in the Colombian Amazon: Ecologically integrating market strategies and subsistence-driven economies? Hum Ecol. 1995;23(3):335–56. 10.1007/BF01190136.

[36] Hecht SB. The evolution of agroecological thought and the case of the Amazon. Cambridge (MA): Harvard University Press; 1989.

[37] Hecht SB. The scramble for the Amazon and the lost paradise of Euclides da Cunha. Chicago (IL): University of Chicago Press; 2013. 10.7208/chicago/9780226322834.001.0001.

[38] Hoffmann WA, Geiger EL, Gotsch SG, Rossatto DR, Silva LCR, Lau OL, Franco AC. Ecological thresholds at the savanna–forest boundary: How plant traits, resources and fire shape tropical landscapes. Ecol Lett. 2012;15(7):759–68. 10.1111/j.1461-0248.2012.01789.x. 22554474 10.1111/j.1461-0248.2012.01789.x

[39] Hoffmann KM, Davis EL, Wickham SB, Schang K, Johnson A, Larking T, Lauriault PN, Le NQ, Swerdfager E, Trant AJ. Conservation of Earth’s biodiversity is embedded in Indigenous fire stewardship. Proc Natl Acad Sci U S A. 2021;118(32):e2105073118. 10.1073/pnas.2105073118. 34362847 10.1073/pnas.2105073118 PMC8364180

[40] Ingold T. The perception of the environment: Essays on livelihood, dwelling and skill. London: Routledge; 2000.

[41] Kimmerer RW. Braiding sweetgrass: Indigenous wisdom, scientific knowledge, and the teachings of plants. Minneapolis (MN): Milkweed Editions; 2013.

[42] Lazzarini L, Neves AL, Falleiro RM. Avaliação da eficácia da implementação de brigadas indígenas como política de combate a incêndios florestais. Biodivers Bras. 2016;6(2):106–20. 10.37002/biodiversidadebrasileira.v6i2.527.

[43] Leonel M. O uso do fogo: O manejo indígena e a piromania da monocultura. Estud Av. 2000;14(40):231–50. 10.1590/S0103-40142000000300019.

[44] Lévi-Strauss C. O cru e o cozido. São Paulo: Cosac & Naify; 1976.

[45] Lévi-Strauss C. Antropologia estrutural II. Rio de Janeiro: Tempo Brasileiro; 1984.

[46] Miguel A. Floresta em chamas, sociedade em degradação: Diagnóstico das políticas ambientais em Rondônia (Amazônia brasileira), 2018–2024. *Rev Emeron.* 2024;3(1):122–45. Available from: https://periodicos.emeron.edu.br/index.php/emeron/article/view/334

[47] Mistry J, Berardi A, Andrade V. Indigenous fire management in the cerrado of Brazil: The case of the Krahô of Tocantins. Hum Ecol. 2005;33(3):365–86. 10.1007/s10745-005-4143-8.

[48] Mistry J, Bilbao B, Berardi A. Community owned solutions for fire management in tropical ecosystems: Case studies from Indigenous communities of South America. Philos Trans R Soc Lond B Biol Sci. 2016;371(1696):20150174. 10.1098/rstb.2015.0174. 27216507 10.1098/rstb.2015.0174 PMC4874412

[49] Mistry J, Berardi A, Schmidt IB. New perspectives in fire management in South American ecosystems. Ambio. 2019;48(5):483–92. 10.1007/s13280-018-1054-7. 10.1007/s13280-018-1054-7 PMC6346601 29752682

[50] Moritz MA, Batllori E, Bradstock RA, Gill AM, Handmer J, Hessburg PF, Leonard J, McCaffrey S, Odion DC, Schoennagel T, Syphard AD. Learning to coexist with wildfire. Nature. 2014;515(7525):58–66. oi.org/10.1038/nature13946. 25373675 10.1038/nature13946

[51] Nepstad DC, Stickler CM, Almeida OT. Globalization of the Amazon soy and beef industries: Opportunities for conservation. Conserv Biol. 2006;20(6):1595–603. 10.1111/j.1523-1739.2006.00510.x. 17181794 10.1111/j.1523-1739.2006.00510.x

[52] Noda SN, Martins ALU, Noda H, Silva AICD, Braga MDS. Paisagens e etnoconhecimentos na agricultura Ticuna e Cocama no alto Rio Solimões, Amazonas. Bol Mus Para Emílio Goeldi Cienc Hum. 2012;7(2):397–416.

[53] Oliveira MR, Falleiro RM, Eloy L, Schmidt IB. Indigenous brigades change the spatial patterns of wildfires and the influence of climate on fire regimes. J Appl Ecol. 2022;59(4):1025–38. 10.1111/1365-2664.14139.

[54] Page MJ, McKenzie JE, Bossuyt PM, Boutron I, Hoffmann TC, Mulrow CD, et al. The PRISMA 2020 statement: An updated guideline for reporting systematic reviews. BMJ. 2021;372:n71. 10.1136/bmj.n71. 33782057 10.1136/bmj.n71 PMC8005924

[55] Pivello VR. The use of fire in the Cerrado and Amazonian rainforests of Brazil: Past and present. Fire Ecol. 2011;7(1):24–39. 10.4996/fireecology.0701024.

[56] Pivello VR. Fire management for biological conservation in the Brazilian Cerrado. In: Belcher CM, editor. Fire phenomena and the Earth system: An interdisciplinary guide to fire science. London: Routledge; 2017. pp. 199–215. 10.4324/9781315243788-6.

[57] Pivello VR, Fidelis A. Introduction: Fire and the South American ecosystems. In: Fidelis A, Pivello VR, editors. Fire in the South American ecosystems. Ecological Studies. Volume 250. Cham: Springer; 2025. pp. 1–8. 10.1007/978-3-031-89372-8_1.

[58] Posey DA. Contact before contact: Typology of post-Columbian interaction with the Northern Kayapó of the Amazon Basin. Bol Mus Para Emílio Goeldi Ser Antropol. 1987;3(2):135–54.

[59] Pyne SJ. Fire: A brief history. 2nd ed. Seattle (WA): University of Washington; 2019.

[60] Rocha MIS, Nascimento DTF. Ocorrência de focos de queimadas em áreas legalmente protegidas do bioma Cerrado (1999/2018). Ateliê Geográfico. 2022;16(2):122–45. 10.5216/ag.v16i2.7080.

[61] Russell-Smith J, Yates CP, Edwards AC, Allan GE, Cook GD, Cooke PM, Craig R, Heath B, Smith R. Contemporary fire regimes of northern Australia, 1997–2011: Frequency, interval, extent, patchiness. Int J Wildland Fire. 2013;22(3):329–41. 10.1071/WF12008.

[62] Santopuoli G, Cachoeira JN, Marchetti M, Viola MR, Giongo M. Explore inhabitants’ perceptions of wildfire and mitigation behaviours in the Cerrado biome, a fire-prone area of Brazil. Ann Silvic Res. 2017;41:29–40. 10.12899/asr-1308.

[63] Sautchuk C, Fagundes GM, Barretto Filho HT. Sociotechnical approach to protected areas and traditional communities. Conserv Biol. 2025;39(3):e70048. 10.1111/cobi.70048. 40444905 10.1111/cobi.70048

[64] Schmidt MVC. *Etnosilvicultura Kaiabi no Parque Indígena do Xingu: subsídios ao manejo de recursos florestais* [dissertação de mestrado]. São Carlos (SP): Escola de Engenharia de São Carlos, Universidade de São Paulo; 2001.

[65] Schmidt MJ. Historical landscape in the Neotropics: A model for prehistoric anthrosol (terra preta) formation in the Upper Xingu. In: Pereira E, Guapindaia V, editors. *Arqueologia Amazônia.* Belém: Museu Paraense Emílio Goeldi (MPEG); Instituto do Patrimônio Histórico e Artístico Nacional (IPHAN); Secretaria de Estado de Cultura do Pará; 2010. pp. 853–78.

[66] Schmidt M, Heckenberger MJ. Amazonian dark earth formation in the Upper Xingu of southeastern Amazonia, Mato Grosso, Brazil. Paper presented at: 71st Annual Meeting of the Society for American Archaeology (SAA); 2006; San Juan, Puerto Rico.

[67] Schmidt IB, Moura LC, Ferreira MC, Eloy L, Sampaio AB, Dias PA, Berlinck CN. Fire management in the Brazilian savanna: First steps and the way forward. J Appl Ecol. 2018;55(5):2094–101. 10.1111/1365-2664.13118.

[68] Silvério DV, Oliveira RS, Flores BM, Brando PM, Almada HK, Furtado MT, Moreira FG, Heckenberger M, Ono KY, Macedo MN. Intensification of fire regimes and forest loss in the Território Indígena do Xingu. Environ Res Lett. 2022;17(4):045012. 10.1088/1748-9326/ac5713.

[69] Susnik B. Etnohistoria de los Guaraníes-Chiriguano. Asunción: Museo Etnográfico Andrés Barbero; 1979.

[70] Toledo FF, Macedo RS. Manejo integrado do fogo no Brasil: Ato lícito no Piroceno / Integrated fire management in Brazil: Lawful act in the Pyrocene / Gestión integrada del fuego en Brasil: Acto legal en el Piroceno. Veredas Dir. 2025;22(1):e223044. 10.18623/rvd.v22.3044.

[71] Viveiros de Castro E. Os pronomes cosmológicos e o perspectivismo ameríndio. Mana. 1996;2(2):115–44. 10.2307/3034157.

[72] Walker WS, Gorelik SR, Baccini A, Aragon-Osejo JL, Josse C, Meyer C, Macedo MN, Augusto C, Rios S, Katan T, de Souza AA, Cuellar S, Llanos A, Zager I, Carvalho L. An analysis of fire dynamics in and around indigenous territories and protected areas in a Brazilian agricultural frontier. Environ Res Lett. 2022;17(9):095010. 10.1088/1748-9326/ac8237.

[73] Welch JR. Xavante ritual hunting: Anthropogenic fire, reciprocity, and collective landscape management in the Brazilian cerrado. Hum Ecol. 2014;42(1):47–59. 10.1007/s10745-013-9637-1.

[74] Welch JR, Coimbra CEA Jr. Indigenous fire ecologies, restoration, and territorial sovereignty in the Brazilian Cerrado: The case of two Xavante reserves. Land Use Policy. 2021;104:104055. 10.1016/j.landusepol.2019.104055.

[75] Welch JR, Brondízio ES, Hetrick SS, Coimbra CEA Jr. Indigenous burning as conservation practice: Neotropical savanna recovery amid agribusiness deforestation in central Brazil. PLoS ONE. 2013;8(12):e81226.10.1371/journal.pone.0081226 . 24349045 10.1371/journal.pone.0081226 PMC3862848

[76] Xerente PPGS, Oliveira RCS. Abordagem indígena sobre manejo integrado do fogo em terras indígenas no Estado do Tocantins – Brasil. Biodivers Bras. 2021;11(2):190–8. 10.37002/biodiversidadebrasileira.v11i2.1719.

